# Comprehensive Evaluation of Injectability Attributes in OxiFree™ Dermal Fillers: MaiLi^®^ Product Variants and Clinical Case Reports

**DOI:** 10.3390/gels10040276

**Published:** 2024-04-19

**Authors:** Patrick Micheels, Alexandre Porcello, Thierry Bezzola, Daniel Perrenoud, Marie-Odile Christen, Lee Ann Applegate, Alexis Laurent

**Affiliations:** 1 Private Medical Practice, CH-1224 Chêne-Bougeries, Switzerland; 2 Development Department, Abcello Sàrl, CH-1432 Belmont-sur-Yverdon, Switzerland; alexandre.porcello@abcello.com; 3 Private Medical Practice, CH-1204 Geneva, Switzerland; tbezzola@gmail.com; 4 Private Medical Practice, CH-1006 Lausanne, Switzerland; drperrenoud@gmail.com; 5 Private Office, F-75116 Paris, France; christen.marieodile3@gmail.com; 6 Regenerative Therapy Unit, Lausanne University Hospital, University of Lausanne, CH-1066 Epalinges, Switzerland; lee.laurent-applegate@chuv.ch; 7 Center for Applied Biotechnology and Molecular Medicine, University of Zurich, CH-8057 Zurich, Switzerland; 8 Oxford OSCAR Suzhou Center, Oxford University, Suzhou 215123, China; 9 Manufacturing Department, TEC-PHARMA SA, CH-1038 Bercher, Switzerland; 10 Manufacturing Department, LAM Biotechnologies SA, CH-1066 Epalinges, Switzerland

**Keywords:** aesthetic medicine, case reports, cross-linked hyaluronic acid, dermal fillers, hydrogel systems, injectability, medical device, needle gauge, OxiFree™ technology, technical benchmarking

## Abstract

Dermal filler injectability is a critical factor for commercial product adoption by medical aesthetic professionals and for successful clinical administration. We have previously reported (in vitro and ex vivo) cross-linked hyaluronic acid (HA)-based dermal filler benchmarking in terms of manual and automated injectability requirements. To further enhance the function-oriented product characterization workflows and the clinical relevance of dermal filler injectability assessments, the aim of this study was to perform in vivo evaluations. Therefore, several variants of the MaiLi^®^ product range (OxiFree™ technology) were characterized in vitro and in vivo in terms of injectability attributes, with a focus on hydrogel system homogeneity and ease of injection. Firstly, standardized in vitro assays were performed in SimSkin^®^ cutaneous equivalents, with variations of the clinical injector, injection site, and injection technique. Then, automated injections in SimSkin^®^ cutaneous equivalents were comparatively performed in a texture analysis setup to obtain fine-granulometry injection force profile results. Finally, five female participants were recruited for the in vivo arm of the study (case reports), with variations of the clinical injector, injection site, and injection technique. Generally, the obtained quantitative force values and injection force profiles were critically appraised from a translational viewpoint, based on discussions around the OxiFree™ manufacturing technology and on in-use specialized clinician feedback. Overall, the present study outlined a notable level of homogeneity across the MaiLi^®^ product range in terms of injectability attributes, as well as consistently high ease of administration by medical aesthetic clinicians.

## 1. Introduction

Dermal filler product injectability attributes are linked to the quality, safety, and efficacy outcomes of specific medical aesthetic interventions [1,2]. Furthermore, the clinical results of facial dermal filler applications are dependent upon the skill and experience level of the practicing physician. Therein, the level of control over the filler injection process is critical to ensure that the correct and desired amount of hydrogel is placed at the appropriate anatomical site and depth [1,3,4]. Importantly, the use of inhomogeneous dermal filler products (i.e., in terms of injectability attributes) may therefore result in excess in vivo product dispensing or in inappropriately shallow gel placement, potentially leading to adverse effects and clinical failure [5,6,7,8,9]. Of note, close consideration of dermal filler product injectability is of central importance for widely used cross-linked hyaluronic acid (HA)-based hydrogel systems. Specifically, their formulation parameters and biophysical attributes may be linked to the overall quality of the provided medical aesthetic care [10,11,12,13,14,15,16].

Notably, previous experimental work by the authors reported some in-use disparities in the injectability attributes of various commercial cross-linked HA-based dermal fillers, within standardized in vitro and ex vivo product benchmarking setups [1]. Specifically, the parallel characterization of 28 dermal fillers (i.e., BELOTERO^®^, JUVÉDERM^®^, VIVACY^®^, RESTYLANE^®^, and TEOSYAL^®^ brands) revealed that inter-product and intra-product qualitative and quantitative variations existed in terms of injectability [1]. Therein, much of the reported intra-brand variability (i.e., as regards injectability attributes) was linked to the specific composition (i.e., HA content, lidocaine presence) or the manufacturing process of individual product variants [1]. Overall, the available experimental data did not enable the identification of a product manufacturer achieving a high level of brand-wide homogeneity and consistency in terms of in vitro and ex vivo hydrogel system injectability [1].

Contrasting with such findings, preliminary in-use clinical feedback by the practicing co-authors reported no tangible discrepancies or differences in the in vivo injection characteristics of several MaiLi^®^ product variants (i.e., MaiLi^®^ Precise, Define, Volume, Extreme; Oxi-Free™ manufacturing technology) [17]. Based on such subjective reports of qualitatively and quantitatively enhanced injectability attributes and building on previous scientific works, prospective investigations of MaiLi^®^ dermal fillers in terms of in-use behaviour were warranted [1,18,19,20,21]. Specifically, a key design interest of the new study was to compare in vitro and in vivo MaiLi^®^ product injectability (i.e., during clinical facial dermal filling) to maximize the translational and clinical relevance of the obtained datasets.

Therefore, the first aim of this study was to perform in vitro qualitative and quantitative injectability attribute assessments on a range of four OxiFree™-based cross-linked HA dermal fillers (i.e., MaiLi^®^ Precise, Define, Volume, and Extreme variants). Therein, standardized in vitro manual injectability evaluations with multi-injector assessments were performed, followed by automated product extrusion in SimSkin^®^ cutaneous equivalents. The second aim of this study was to transpose the manual product injectability assessments in vivo, under real-world in-use clinical conditions. Therefore, dynamometric evaluations of MaiLi^®^ dermal filler injection forces were performed by two specialized clinicians during patient treatment and were analysed in the form of five clinical case reports. 

Of note, the primary hypothesis of the study was that MaiLi^®^ dermal fillers present intra-product and brand-wide homogeneity as regards their injectability attributes. Specifically, it was hypothesized that very smooth injection force plateaus could be obtained when injecting MaiLi^®^ products, with low quantitative differences between the product variants. The secondary hypothesis of the study was that MaiLi^®^ dermal fillers present an enhanced quality level of injectability attributes as compared to similar medical devices. Specifically, it was hypothesized that the MaiLi^®^ product brand could display better felt behaviours (i.e., by the physician) during administration and smoother injection force plateaus than alternative dermal fillers. Overall, the originality/novelty and significance of the study resided in the use of advanced and multiparametric quantitative setups (i.e., in vitro and in vivo) for experimental commercial dermal filler product injectability attribute characterization, enabling detailed technical investigation and providing enhanced translational relevance. Notably, the dual use of standardized in vitro setups for product injectability characterization and the in vivo measurement of product injection forces was uniquely brought forth herein for optimal demonstration of the key features of the OxiFree™ technology under real-world conditions. Such an approach is of high relevance and informative value for clinical practitioners, supplementing manufacturer-provided data. Namely, the reported results significantly illustrated the importance of selecting high-quality aesthetic devices and care centres with track records of clinical expertise to obtain the highest level of medical aesthetic performance. 

## 2. Results and Discussion

### 2.1. Technical Benchmarking of MaiLi^®^ Dermal Filler Product Attributes

The retained investigational products consisted of four CE-marked dermal filler variants (i.e., Precise, Define, Volume, Extreme) of the MaiLi^®^ brand (Sinclair Pharma Ltd., London, UK), exclusively based on the OxiFree™ HA cross-linking technology [22,23]. Prior to the experimental investigations, brief technical documentary reviews were performed based on the available manufacturer-provided elements for preliminary general product attribute benchmarking (Table 1). 

Of note, all of the considered MaiLi^®^ dermal filler variants are industrially manufactured using a specifically designed hydrogel production process (Table 1). Namely, enhanced hydrogel system resilience and distinctive projection capacities were previously reported for OxiFree™-based products compared to alternative commercial dermal fillers [19,20]. Specifically, such attributes are derived through an optimized manufacturing process, which may be broken down into four main phases, as follows [23]:

(a) Hydration of high molecular weight, non-animal origin, pharmaceutical grade HA;
(b) Sparing BDDE-based HA chemical cross-linking under a protective atmosphere for viscoelastic hydrogel formation;
(c) Extraction of reactive oxygen molecules prior to hydrogel product terminal sterilization;
(d) Hydrogel product terminal sterilization in oxidant-deprived conditions.

In addition, noteworthy clinical advantages were previously set forth around this dermal filler technology, comprising the sparing use of hydrogel for patient treatment, based on the enhanced functional parameters conferred by specific viscoelasticity attributes [18,19,20,21]. From a formulation viewpoint, it should be noted that manufacturers are legally required to declare the concentration of HA in their dermal filler products (Table 1). However, they are not required to disclose the molecular weight of the HA raw material nor the degree of cross-linking of the system in the finished product. Therefore, within the same technology or filler product range, manufacturers can modulate the degrees of polymer cross-linking or use multiple HA molecular weight ranges [1]. Specifically, to facilitate product injection, it is possible to incorporate an additional phase of linear HA within a cross-linked polymer system. Thus, such formulation- and processing-based elements allow for finished product technical optimization in order to achieve the desired injectability attributes.

### 2.2. Rheological Characterization of the MaiLi^®^ Dermal Filler Product Range

**Figure 1:**
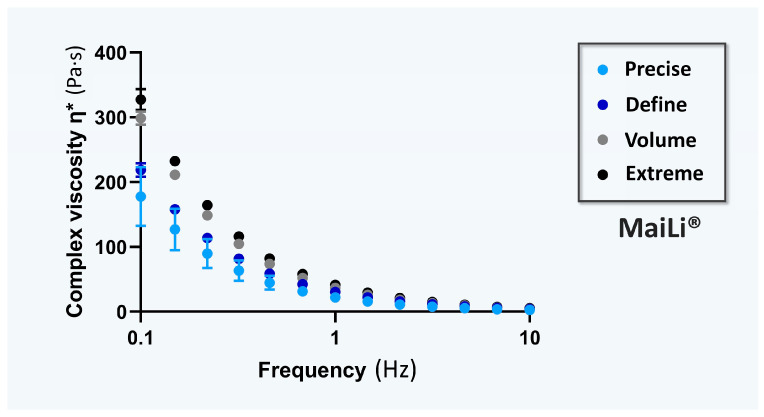
Rheological characterization results of the four MaiLi^®^ dermal filler variants. Complex viscosity data are plotted as means with standard deviations as error bars. Hz, Herz; Pa·s, Pascal seconds.

For the specific comparative documentation of the viscoelasticity attributes of the investigated hydrogel systems, the rheological behaviours of the four MaiLi^®^ product variants were experimentally determined. Specifically, complex viscosity η* values (i.e., in oscillatory rheology) were determined for each dermal filler variant as a function of the applied oscillation frequency (Figure 1).

Of note, complex viscosity (η*) is a measure of a material’s mechanical resistance to deformation under shear stress. It is obtained by calculating the quotient of the maximum stress amplitude and maximum strain rate amplitude [2]. This rheological attribute is classically used to describe the behaviour of HA-based fillers in terms of “thickness” or “resistance to flow” during hydrogel injection [2,24,25,26]. Namely, viscosity appears to better quantitatively approximate HA-based dermal filler injectability when it is interpreted as the process of extrusion from the syringe/needle and for immediate tissue integration [10]. 

Of further note, a material with a low η* value is easy to deform, and conversely, a high η* value is characteristic of a material which is more difficult to deform. By extension, a low η* value suggests that the considered dermal filler is easier to inject, whereas a high η* value means that the hydrogel product is more challenging to inject, with a higher shear thinning point and yield stress values [6,10,11]. Interestingly, the experimental rheological data gathered in this study enabled the classification of the MaiLi^®^ product variants based on decreasing mean complex viscosity η* values (i.e., Extreme > Volume > Define > Precise; Figure 1). Such results confirmed the technical validity of the specified indications/intended product uses for the MaiLi^®^ product variants (i.e., ranging from powerful filling and facial sculpting to skin-finishing for smoothing wrinkles; Figure 1, Table 1). 

Of final note, while hydrogel system rheological properties may be a useful technical proxy for finished product injectability during preliminary screening assays, it is important to take formulation and packaging/accessory specifics into consideration for product injectability characterization. For example, MaiLi^®^ Precise and MaiLi^®^ Define are both meant to be injected through a 30 G × ½″ needle, yet the HA concentration of the Precise variant is 3.0 mg/mL lower than that of the Define variant (i.e., 15.0 mg/mL against 18.0 mg/mL, respectively; Table 1). Therefore, differences in mean and peak injection forces may be expected for these two product variants based on their differential formulation-based specifics (Table 1). Overall, only experimental setups incorporating the final product packaging and the specified administration system(s) may be considered pertinent for sound injectability attribute characterization.

### 2.3. Manual Injectability Assessments of the MaiLi^®^ Product Range in SimSkin^®^ Cutaneous Equivalents

The identified relevance of performing manual injectability evaluations of dermal fillers is based on the low informative value of product extrusion forces (i.e., automatic protocols, extrusion in atmospheric air), generally used as functional characteristics or as features by product manufacturers [1]. Specifically, it was previously noted that the manual injection forces of cross-linked HA-based dermal fillers were systematically inferior to the values obtained in automated laboratory settings [1]. 

For the needs of the present study, each MaiLi^®^ product variant was injected in vitro at the indicated anatomic depth (i.e., intradermal or hypodermal injections in SimSkin^®^ substrates, Figure S1) by three practicing physicians. Among the three operators, only co-author PM had clinical experience with the use of MaiLi^®^ products, whereas co-authors TB and DP were initially not familiar with this medical device brand. Additionally, based on off-label (i.e., yet widespread) clinical practice of hypodermal injections with wrinkle-filling products, the latter were injected in the artificial hypodermis component of the SimSkin^®^ substrate (Figure S1).

Overall, the three operators each performed four injection regimens for each MaiLi^®^ product variant (i.e., intradermal point-by-point and retro-tracing injections, hypodermal bolus and retro-tracing injections, Figure S1) [1]. For the technical needs of this study, the plunger rods from the MaiLi^®^ dermal filler variants were replaced by those of TEOSYAL RHA^®^ 2 dermal fillers (TEOXANE, Geneva, Switzerland) comprising a hilt-mounted dynamometric sensor [1]. All injections were performed in triplicate by each operator and for each MaiLi^®^ product variant, where the mean forces of injection were recorded (Table 2, Table 3, Table 4 and Table 5).

For the MaiLi^®^ Precise variant, the corresponding in vitro injection force profiles were presented in Figure S2 (Table 2). Overall, while the injection force curves were found to be consistent and similar in profile across all three injectors, the recorded mean values were systematically higher for injector TB (Table 2, Figure S2). It is noteworthy that injector DP administered the product rapidly in all experimental settings as compared to injectors PM and TB. The experimental results obtained for the MaiLi^®^ Define variant were on average higher in value than for the MaiLi^®^ Precise variant and were found to be less dispersed between injectors (Table 2 and Table 3).For the MaiLi^®^ Define variant, the corresponding in vitro injection force profiles were presented in Figure S3 (Table 3). For this hydrogel, injector DP administered the product rapidly again, with systematically and surprisingly lower mean injection forces as compared to injectors PM and TB (Table 3). For the MaiLi^®^ Volume variant, high inter-injector variability was noted for the measured injection forces, yet low inter-injection site variability was noted for each injector (Table 4).

For the MaiLi^®^ Volume variant, the corresponding in vitro injection force profiles were presented in Figure S4 (Table 4). It should be noted that the MaiLi^®^ Volume product is not indicated for intradermal injection, as it is primarily destined for hypodermal or close-to-the-bone placement (Table 1). Notably, it was observed that hypodermal bolus injections by injector PM were comparatively less homogeneous and required more force, which was attributed to the use of the P1–P2 thumb joint for the injections. Additionally, these relatively high force values were correlated with the lower clinical experience level of injector PM with volumizing agents (Figure S4). Here again, injector DP administered the product relatively rapidly and with low overall mean injection forces (Table 4, Figure S4). Finally, as concerns the MaiLi^®^ Extreme variant, the experimental injection force values were found to be closer between injectors PM and DP, with the values of injector TB being systematically recorded as higher (Table 5). 

For the MaiLi^®^ Extreme variant, the corresponding in vitro injection force profiles were presented in Figure S5 (Table 5). Here again, it was noted that the considered hydrogel product is not indicated for hypodermal injection (Table 1). In this case, it was set forth that the injection forces exerted by injector TB could possibly be superior to those of injector PM because of the use of the thenar eminence for plunger rod actuation by the latter (Table 5). Furthermore, the injection force curves were found to be notably more variable on average for the MaiLi^®^ Extreme product variant, as compared to the other three MaiLi^®^ product variants (Figures S2–S5).

Generally, the recorded inter-injector variability as regards in vitro product injection forces was attributed to varying levels of experience with the MaiLi^®^ product range, varying speeds of injection, and the use of different thumb portions by the injectors (Table 2, Table 3, Table 4 and Table 5, Figures S2–S5). Specifically, it was previously reported that the use of the P1-P2 thumb joint or the thenar eminence generally resulted in superior mean injection forces as compared to the use of the thumb pulp [1].

As regards the off-label injection of the various MaiLi^®^ product variants, such approaches were experimentally performed based on real-world clinical practice and in view of methodological continuity with previous reports by the authors [1,17]. Generally, it was noted that the recorded injection forces were relatively low as compared to manufacturer-provided MaiLi^®^ injectability data [22]. Finally, the recorded injection force profiles were assessed to be qualitatively consistent across the considered MaiLi^®^ product range and for all three clinical injectors (Figures S2–S5). Importantly, such elements suggested that hydrogel systems based on the OxiFree™ technology possess high levels of intra-product homogeneity, contrasting with some declinations of the BELOTERO^®^ or TEOSYAL^®^ product ranges [1]. 

### 2.4. Automated In Vitro MaiLi^®^ Product Injectability Assessments: Comparative Injection Force Curves for Standardized Dermal Filler Product Benchmarking

**Figure 2:**
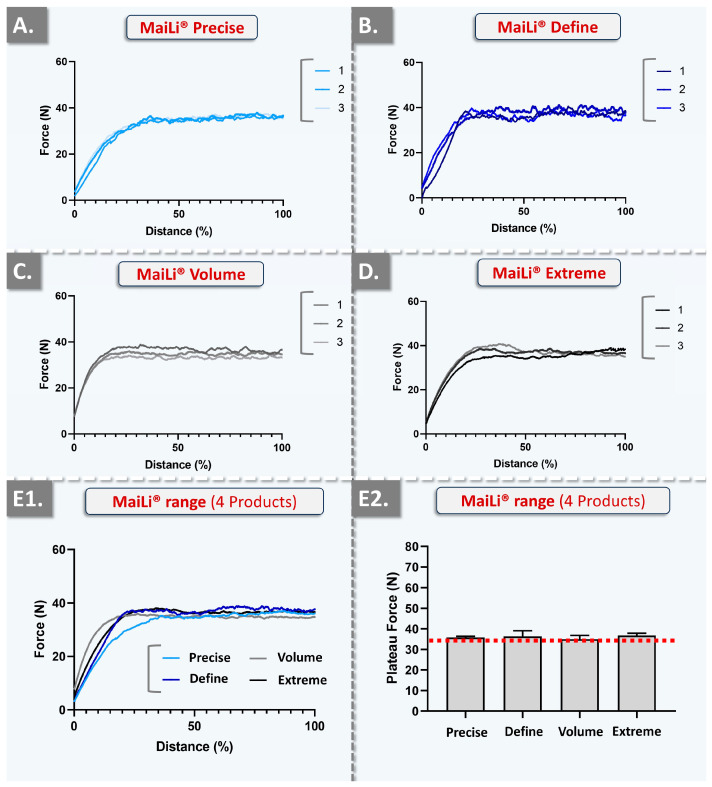
Experimental results of automated injectability evaluations at a constant plunger rod actuation speed of 1 mm·s^−1^ with MaiLi^®^ product in vitro injection in SimSkin^®^ cutaneous substrates. (**A**) Injection force curves of the MaiLi^®^ Precise product. (**B**) Injection force curves of the MaiLi^®^ Define product. (**C**) Injection force curves of the MaiLi^®^ Volume product. (**D**) Injection force curves of the MaiLi^®^ Extreme product. (**E1**) Combined mean injection force curves of the four MaiLi^®^ product variants. (**E2**) Comparison of the mean injection force plateaus of the four MaiLi^®^ product variants. The overall mean plateau injection force (i.e., dotted red line) was found to be 35 N (Table 6). N, Newtons.

**Figure 3:**
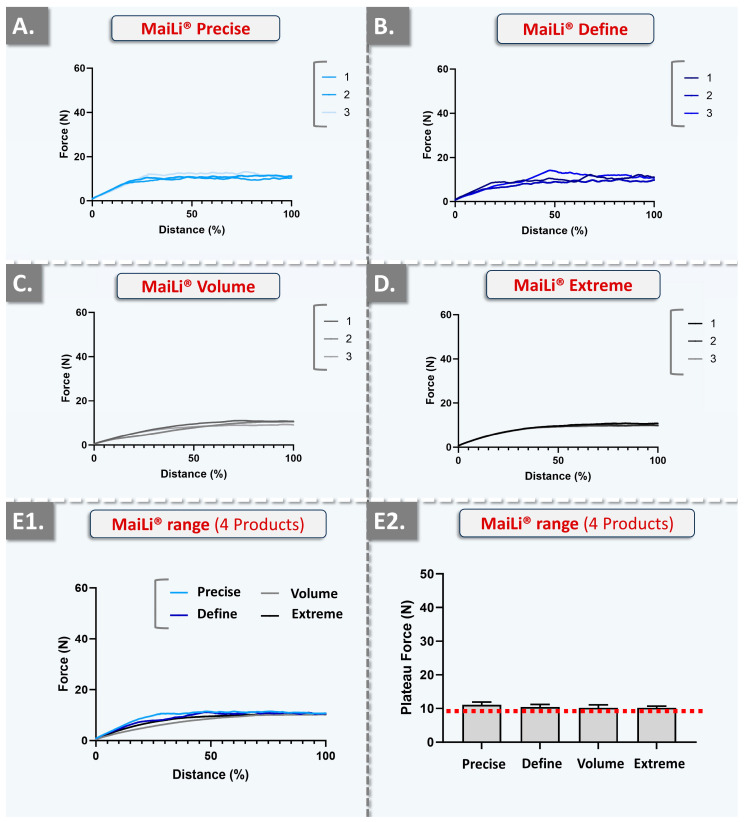
Experimental results of automated injectability evaluations at a constant plunger rod actuation speed of 0.2 mm·s^−1^ with MaiLi^®^ product in vitro injection in SimSkin^®^ cutaneous substrates. (**A**) Injection force curves of the MaiLi^®^ Precise product. (**B**) Injection force curves of the MaiLi^®^ Define product. (**C**) Injection force curves of the MaiLi^®^ Volume product. (**D**) Injection force curves of the MaiLi^®^ Extreme product. (**E1**) Combined mean injection force curves of the four MaiLi^®^ product variants. (**E2**) Comparison of the mean injection force plateaus of the four MaiLi^®^ product variants. The overall mean plateau injection force (i.e., dotted red line) was found to be 10 N (Table 6). N, Newtons.

In order to gain further insights into the injectability attributes of the considered MaiLi^®^ product variants (i.e., and to enhance the qualitative and quantitative levels of product characterization), in vitro automated injections were performed. Therein, repeated texture analysis measurements enabled us to visualize and quantify the plateau forces of product injection in SimSkin^®^ cutaneous equivalents at two specified and constant injection speeds (Figure 2 and Figure 3, Table 6). 

Two highly interesting aspects were noted about the automated injection force profiles of the considered MaiLi^®^ dermal filler variants. Firstly, from a qualitative standpoint, the very high degree of force plateau smoothness (i.e., as observed across all groups) was assessed as specifically informative regarding intra-product system homogeneity in terms of viscoelasticity and general biophysical attributes (Figure 2 and Figure 3) [1]. Additionally, the high reproducibility of the analyses (i.e., superposed injection force curves for the experimental replicates and across all groups) denoted inter-syringe and brand-wide system homogeneity, despite the respective formulation-related specificities (Table 1, Figure 2 and Figure 3). 

Importantly, the gathered in vitro automated injection force data were found to clearly stand out as compared to the profiles which were previously gathered for BELOTERO^®^ or TEOSYAL^®^ product variants [1]. Therein, some BELOTERO^®^ (e.g., lidocaine-free Balance variant) or TEOSYAL^®^ (e.g., RHA^®^ 1, Ultra Deep^®^) groups were found to present inter-variant variability and intra-syringe inhomogeneity (i.e., in terms of injectability) [1]. Of highest importance, the obtention of superposing injection force curves in an automated setup for all the variants of a product brand (i.e., MaiLi^®^ Precise, Define, Volume, and Extreme) was interpreted as extremely technically interesting, contrasting with previous commercial product screenings (Figure 2 and Figure 3) [1]. Furthermore, from a quantitative standpoint, it was noted that the mean plateau injection forces were highly consistent between the experimental groups (Figure 2(E2) and Figure 3(E2), Table 6).

Such closely distributed experimental data underscored the tight control over MaiLi^®^ product biophysical attributes during the hydrogel manufacturing phases (Table 6). Of note, as regards the quantitative levels of injection force for the MaiLi^®^ product variants, the automated in vitro setup yielded significantly higher values than the manual in vitro setups (Table 2, Table 3, Table 4, Table 5 and Table 6). In detail, the relatively rapid and constant injection speed of 1 mm·s^−1^ was used herein for optimal data comparability with previous reports, yet such speeds are not common in clinical practice (i.e., even for bolus injections) [1]. Therefore, the slower injection speed of 0.2 mm·s^−1^ (i.e., closer to that of clinical practice) was additionally used herein and confirmed that the hydrogel injection force is positively correlated to the injection speed in an automated setup (Table 6). 

Specifically, the obtained automated injectability results were found to be systematically higher in value than those obtained in the various iterations of the manual setup (i.e., 10–35 N versus 0.50–2.00 N, respectively; Table 2, Table 3, Table 4, Table 5 and Table 6). Importantly, the quantitative difference between the automated and manual injectability setups, in terms of injection force requirements, may be attributed to the differing injection geometries. Specifically, the automated setup comprised the perpendicular insertion of the needle in the SimSkin^®^ cutaneous equivalent, whereas the manual injections were performed using needle angles of 10–19° with the skin plane (i.e., the most obtuse angles being used for hypodermal bolus injections). 

Overall, the key clinical interest of obtaining consistent injection forces across an entire product range is to optimize the in-use experience of the injector with a given dermal filler technology [1]. In detail, while various MaiLi^®^ product variants exist and are finely tuned in terms of biophysical attributes (i.e., for differential clinical indications), conserved injection forces may be used for each variant (Table 2, Table 3, Table 4, Table 5 and Table 6). The resulting direct advantage for the clinician is that all MaiLi^®^ product variants should behave the same in terms of injectability in defined settings (Figure 2 and Figure 3). Thus, a high level of consistency of in-use product behaviour may be anticipated, thereby conferring optimal control over the administration process to the injector. As previously mentioned, this aspect is to be considered as a critical component (i.e., for optimizing patient experience) of the provided medical aesthetic care quality [1]. 

### 2.5. Clinical Case Reports on In Vivo MaiLi^®^ Dermal Filler Injectability during Patient Treatments

In order to finally provide data with the highest level of clinical relevance, in vivo quantitative product injectability assessments were performed on five female participants. In detail, consenting participants were included in the study and were treated with MaiLi^®^ product variants by injector PM or by injectors PM and TB in the cases where the participant accepted treatment by two different injectors (i.e., one injector per face side). The anonymized in vivo study parameters are summarized in Table 7.

**Figure 4:**
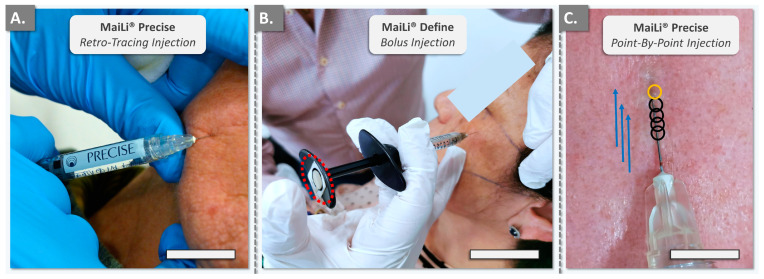
Photographic records of the clinical case reports for in vivo MaiLi^®^ dermal filler product injectability assessment. (**A**) Injection force was applied with the thenar eminence of the thumb. Scale bar = 20 mm. (**B**) Injection force was applied with the thumb pulp. The dynamometric sensor is visible on the plunger rod hilt (i.e., dotted red outline). Scale bar = 30 mm. (**C**) Illustration of the point-by-point injection technique methodology. Black circles illustrate injections which were already realised, where the bevel of the needle was introduced in the centre of the circle. The area of the next injection is outlined in orange. The directionality and direction of progression are depicted by blue arrows. Additional methodological details are presented in Figure S6. Scale bar = 6 mm.

The retained MaiLi^®^ dermal filler injection techniques corresponded to those routinely used in the clinical practice of injector PM for facial wrinkle filling and volumetric correction (Table 7). Importantly, it should be noted that the in vivo portion of the study only focussed on the filler administration-related injectability measurements (i.e., quantitative force values) and did not focus on efficacy-related outcomes. The various MaiLi^®^ products were injected using either the thenar eminence, the pulp, or the P1–P2 joint of the thumb (Figure 4). 

It is of note that the FlexiForce^®^ dynamometric sensor and the attached cable may have slightly modified the position of the product syringe in the hand of the injector, as compared to routine clinical practice (Figure 4B). Generally, the recorded in vivo manual injection forces were found to be inferior in value to the data gathered in vitro in the automated setup, in adequation with the in vitro manual data (Table 2, Table 3, Table 4, Table 5 and Table 6). Furthermore, quantitative differences were observed between the injectors (i.e., injector PM was well experienced with MaiLi^®^ products, injector TB was a new user) and between the areas of the thumb used for the injections (i.e., higher forces for the P1–P2 joint compared to the thumb pulp; Figure 4).

#### 2.5.1. First Case Report: In Vivo MaiLi^®^ Precise Injectability Assessments for Participant N°1

**Figure 5:**
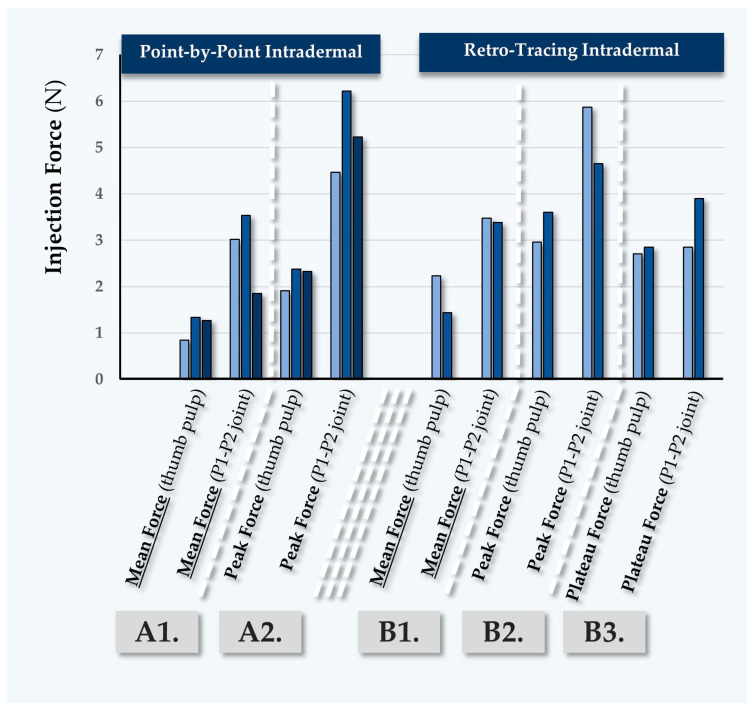
Quantitative results of in vivo dermal filler product injectability evaluation in Participant N°1, using the MaiLi^®^ Precise dermal filler. Mean forces (A1) and peak forces (A2) were plotted for intradermal point-by-point injections. Mean forces (B1), peak forces (B2), and plateau forces (B3) were plotted for intradermal retro-tracing injections. Selected injection force data and injection force profiles are presented in Figures S7–S9 and in Table S1. N, Newtons.

Participant N°1 was treated for general wrinkle filling of the face, using the MaiLi^®^ Precise dermal filler (Table 7). The recorded forces for point-by-point injection were found to be close between injectors PM and TB (Figure 5, Table S1).

The recorded forces were found to be systematically higher in this case when the P1-P2 joint of the thumb was used (Figure 5). Of note, injector PM performed antero-grade injections using the thumb pulp, where the recorded forces were slightly inferior to those recorded during point-by-point injections (Table 7 and Table S1). As this type of injection is less common for facial wrinkle filling, it is possible that slower injection speeds were used, contributing to the lower recorded injection forces (Table S1). As concerns the retro-tracing injections, higher values were recorded for injector TB, which may be attributed to superior injection speeds as compared to injector PM (Table S1). 

#### 2.5.2. Second Case Report: In Vivo MaiLi^®^ Define Injectability Assessments for Participant N°2 

**Figure 6:**
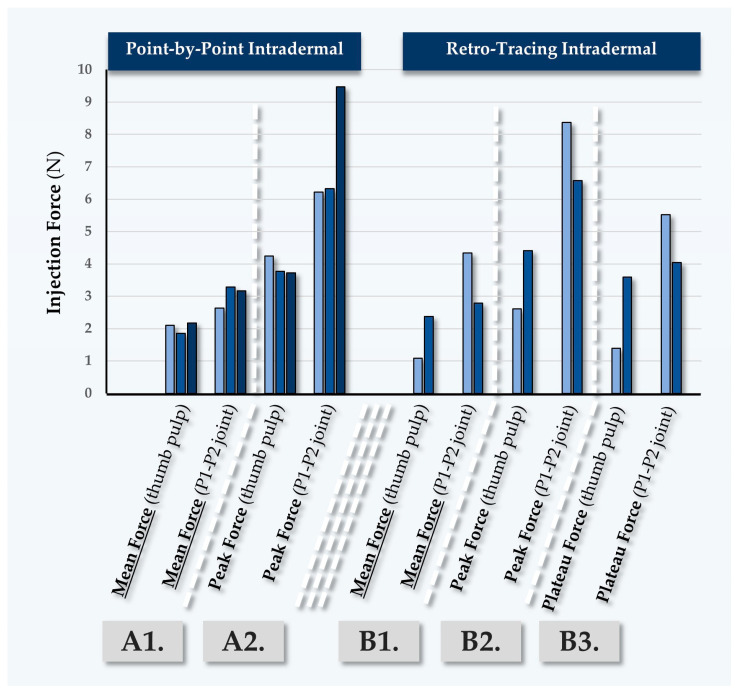
Quantitative results of in vivo dermal filler product injectability evaluation in Participant N°2, using the MaiLi^®^ Define dermal filler. Mean forces (A1) and peak forces (A2) were plotted for intradermal point-by-point injections. Mean forces (B1), peak forces (B2), and plateau forces (B3) were plotted for intradermal retro-tracing injections. Selected injection force data and injection force profiles are presented in Figures S7 and S9 and in Table S2. N, Newtons.

Participant N°2 was treated for general wrinkle filling of the face, using the MaiLi^®^ Define dermal filler (Table 7). Point-by-point and retro-tracing injections were performed by injector PM, yielding low quantitative force values (Figure 6, Table S2).

Here again, the peak injection forces were observed to be high as compared to the mean injection forces (Figure 6). Conversely, the mean injection forces for point-by-point injections were recorded as relatively low (e.g., 1.45 N to 1.51 N; Table S2). Such values were linked to careful and slow injection in the glabella zone, which comports increased risks of adverse intravascular administration as compared to other zones of the face (Table S2). Of note, the antero-tracing and retro-tracing injections required relatively high injection forces as compared to the point-by-point injections. Here again, the higher values recorded for injector TB most probably resulted from higher injection speeds (Table S2).

#### 2.5.3. Third Case Report: In Vivo MaiLi^®^ Volume Injectability Assessments for Participant N°3 

**Figure 7:**
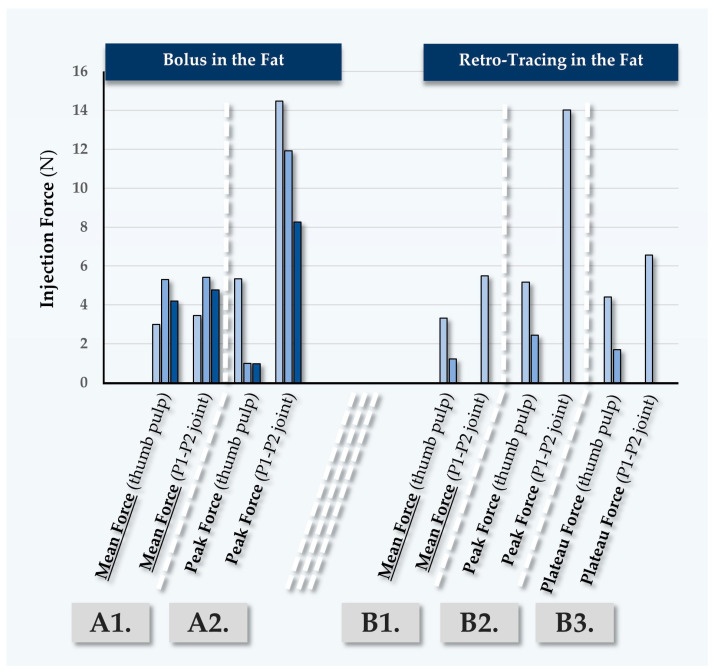
Quantitative results of in vivo dermal filler product injectability evaluation in Participant N°3, using the MaiLi^®^ Volume dermal filler. Mean forces (A1) and peak forces (A2) were plotted for bolus injections in the fat. Mean forces (B1), peak forces (B2), and plateau forces (B3) were plotted for retro-tracing injections in the fat. Selected injection force data and injection force profiles are presented in Figure S10 and in Table S3. N, Newtons.

Participant N°3 was treated for deep wrinkle filling of the face, using the MaiLi^®^ Volume dermal filler (Table 7). The recorded forces for point-by-point injections were found to be close between injectors PM and TB (Figure 7, Table S3).

In this case, various injection depths were used (Table 7). The recorded injection forces were found to be significantly higher than those observed in previous participants, due to the fact that the injections were performed in the fat (Figure 7, Table S3). Confirming previous observations, the peak forces which were exerted using the P1-P2 thumb joint were again found to be higher in value than those exerted using the thumb pulp (Figure 7). It is noted that off-label injection in the deep reticular dermis or at the dermal-hypodermal junction is sometimes performed, yet this is only technically possible in zones of thick skin such as the nasogenian folds.

#### 2.5.4. Fourth Case Report: In Vivo MaiLi^®^ Extreme Injectability Assessments for Participant N°4

**Figure 8:**
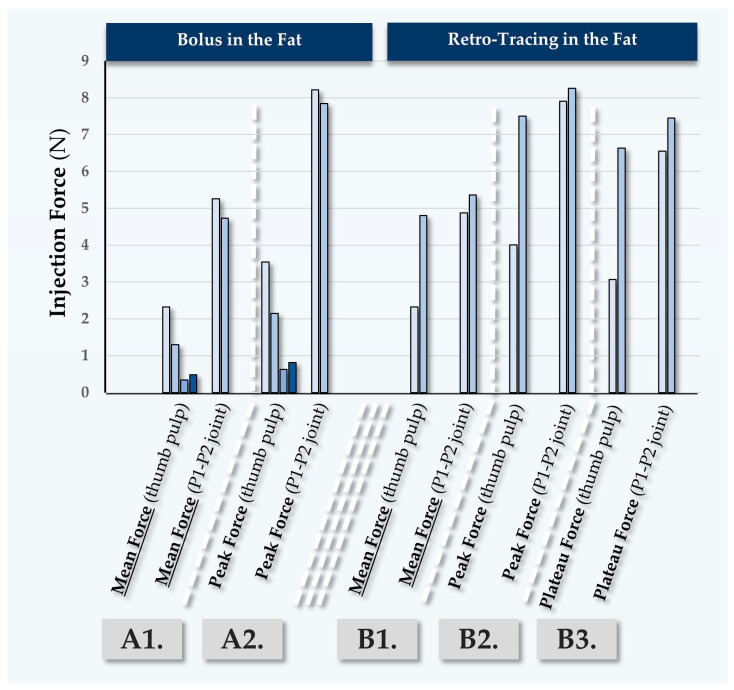
Quantitative results of in vivo dermal filler product injectability evaluation in Participant N°4, using the MaiLi^®^ Extreme dermal filler. Mean forces (A1) and peak forces (A2) were plotted for bolus injections in the fat. Mean forces (B1), peak forces (B2), and plateau forces (B3) were plotted for retro-tracing injections in the fat. Selected injection force data and injection force profiles are presented in Figure S10 and in Table S4. N, Newtons.

Participant N°4 was treated by injector PM for volumizing of the face, using the MaiLi^®^ Extreme dermal filler (Table 7, Figure 8).

In this case, the MaiLi^®^ Extreme dermal filler was injected close-to-the-bone, for the obtention of maximal volumizing effects (Table 7). Highly significant differences were noted in terms of the recorded injection force between injection modalities, where the use of the P1-P2 thumb joint produced excessive injection forces (Table S4). Similarly to the first three clinical cases, high recorded peak injection forces were noted (Figure 5, Figure 6, Figure 7 and Figure 8). 

#### 2.5.5. Fifth Case Report: In Vivo MaiLi^®^ Extreme Injectability Assessments for Participant N°5

**Figure 9:**
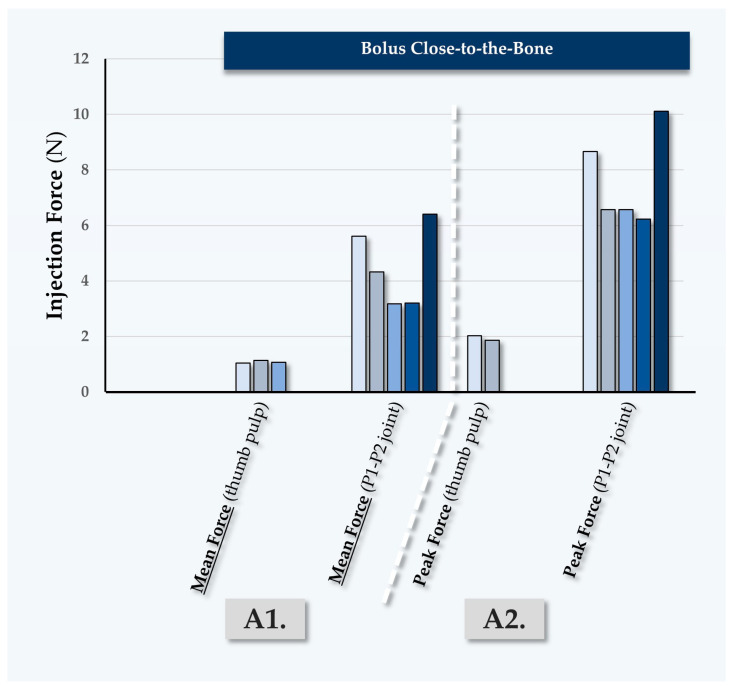
Quantitative results of in vivo dermal filler product injectability evaluation in Participant N°5, using the MaiLi^®^ Extreme dermal filler. Mean forces (A1) and peak forces (A2) were plotted for close-to-the-bone bolus injections. Selected injection force data are presented in Table S5. N, Newtons.

Participant N°5 was treated for volumizing of the face, using the MaiLi^®^ Extreme dermal filler (Table 7, Figure 9). 

In this case, the MaiLi^®^ Extreme dermal filler was injected close-to-the-bone by injector PM, using the thumb pulp and a bolus regimen (Figure 9). Therein, varying injection speeds were used and a positive trend between the injection speed and injection force was evidenced (Table S5). Specifically, the use of rapid injection regimens resulted in peak or plateau injection forces which were approximately double in value as compared to slower injections (Table S5). Furthermore, significantly higher injection forces were recorded with P1-P2 joint injections as compared to thumb pulp injections, which was consistent with the previous findings of the study. 

### 2.6. Clinical Considerations on In Vitro and In Vivo MaiLi^®^ Dermal Filler Injectability

Global consideration of the presented experimental results has enabled us to confirm the primary and secondary hypotheses of the study. Namely, it was specifically shown that MaiLi^®^ product variants present intra-product and inter-product homogeneity as regards their injectability attributes (Figure 2 and Figure 3, Table 6). Furthermore, it was set forth that MaiLi^®^ dermal fillers present enhanced injectability attributes (i.e., smooth and consistent automated injection force plateaus) as compared to similar medical devices [1]. Overall, the gathered data were interpreted to concur with the available clinical feedback on the seamless injectability of MaiLi^®^ dermal fillers, contrasting with that of alternative brands (e.g., specific BELOTERO^®^ or TEOSYAL^®^ variants) [1,17].

The significance of the present study resided in the novelty of reporting in vivo dermal filler in-use injectability assessments, as well as the documentation of the distinctive injectability behaviour of MaiLi^®^ products (Figure 2, Figure 3 and Figure 4). In detail, the presented automated injection force profiles were interpreted to qualitatively stand out from those of five commercial competitor brands (i.e., BELOTERO^®^, JUVÉDERM^®^, VIVACY^®^, RESTYLANE^®^, and TEOSYAL^®^), as assessed by the same operators in the same experimental setups [1]. Therein, no other product manufacturer was found to attain the quality level of the MaiLi^®^ product brand in terms of plateau injection force smoothness and inter-product injectability consistency (Figure 3, Table 6) [1]. 

Importantly, hydrogel product injectability attributes may be studied in diverse experimental setups and are often leveraged by manufacturers as technical features (i.e., to optimize clinician and patient experience of care) [27,28,29,30]. Notwithstanding, tangible correlations may be made between the quality level of dermal filler product injectability and the safety or efficacy outcomes of the treatment [1]. In the case of a homogeneous hydrogel system presenting smooth injection force plateaus (e.g., MaiLi^®^ variants), the clinician may expect no or low levels of jerking/indentation-like behaviour of the plunger rod during filler administration (Figure 3 and Figures S2–S5). Thereby, the risk of clinically injecting excess hydrogel amounts or of shallow product placement (i.e., potential nodule creation or Tyndall effect) is diminished [31,32].

From a quantitative standpoint, the comparative analysis of MaiLi^®^ hydrogel product manual injection force profiles between SimSkin^®^ cutaneous equivalents and human participants showed similarities for all four product variants (Figures S2–S5 and S7–S10). Furthermore, qualitative aspects of hydrogel product behaviour upon injection in both conditions were largely conserved, confirming the high translational relevance of the in vitro SimSkin^®^ model (Figures S7–S10). Notwithstanding, several force values were recorded as being higher in vivo, especially for peak values (Figure 5, Figure 6, Figure 7, Figure 8 and Figure 9, Tables S1–S5). Such results were considered to be mainly linked to the differences in composition and topography between the artificial SimSkin^®^ constructs and human skin [33,34,35,36,37]. Specifically, while differential layers are present in SimSkin^®^ substrates, it is probable that the synthetic material does not exactly match the diversity and nuances of human skin structures (i.e., epidermis, dermis, and hypodermis) and their biomechanical attributes [33,35]. 

Of further note, the presented in vivo data of MaiLi^®^ product injectability also confirmed the clear trend of lower injection forces as compared to the automated in vitro injectability setup (Table 6, Figure 5, Figure 6, Figure 7, Figure 8 and Figure 9). Specifically, these results were interpreted to be mainly linked to the relatively low manual injection speeds used in clinical settings (e.g., 0.05–0.1 mm·s^−1^) as compared to the investigated automated injection speeds (i.e., 0.2–1.0 mm·s^−1^, Table 6). Furthermore, significant inter-injector quantitative differences were recorded as regards MaiLi^®^ product manual injectability (Table 2, Table 3, Table 4 and Table 5). Confirming previous assessments, the present experiments outlined the significant influence of the injection speed, syringe handling modalities (i.e., injection with different portions of the thumb), administration site, and physician experience with a given product on the effective injection force [1]. Finally, it was shown that the depth of injection largely dictated the practically required injection forces of MaiLi^®^ dermal fillers (Table 2, Table 3, Table 4 and Table 5). Specifically, in vivo superficial reticular dermis injections generally required more force than deeper injections, in all probability due to the higher fibre density [33]. However, the experimental values gathered for in vivo MaiLi^®^ injections in the fat were notably higher than those obtained in vitro for hypodermal injections (Figures S2–S5, Tables S1–S5).

Overall, the reported homogeneity of MaiLi^®^ products ensured consistent force levels during injection, addressing previous concerns about in-use pressure variations with alternative filler products [1]. Such attributes may lead to smoother, more uniform aesthetic results, potentially reducing complications like the Tyndall effect or unevenness. Notwithstanding, while the study’s in vitro analyses and the comparisons with previous observations of 28 HA gels offered valuable insights, an important gap between laboratory conditions and clinical practices was recognized [1]. Notably, most manufacturer-provided data, including product extrusion force curves, are usually obtained under conditions which are not fully representative of actual treatment scenarios. Namely, injections are often performed in the air rather than in skin or skin analogues. Therefore, the use of relevant in vitro and in vivo experiments may provide enhanced clinical predictability of the behaviour of dermal filler products [1]. Furthermore, expanding research through multicentre studies involving diverse patient populations and multiple skin types would help to validate the presented laboratory findings in a broader clinical context.

### 2.7. Hydrogel System Biophysical Attributes and Injection Technical Specifications Are Key Modulators of Product Injectability and Performance

In theory, the extrusion force required to push the dermal filler through the needle is mainly impacted by the viscosity of the system. Specifically, higher-viscosity fillers will require more force to extrude, while lower-viscosity fillers will require less force. During the first part of the injection process (i.e., elastic regime), the slope of the injection force curve is proportional to the G′ storage modulus of the system [38]. Then, beyond a yield point, the gel begins to flow and the viscous regime dominates. Depending on the homogeneity of the hydrogel system, the injection of the filler is smooth and occurs at a steady rate [38]. Generally, a close relationship exists between the injection force profiles and clinical outcomes [1]. If the clinician suddenly pauses and stops applying a steady force at any point during the injection, the entire process must be restarted to reach the viscous regime once more. Thus, starting and stopping can lead to an uneven distribution of the product, resulting in an undesirable and uneven outcome. The same issues can be observed in cases of non-homogeneity during the viscous regime [1,38].

From a clinical standpoint, the relationship between injection speed, geometry, and injection force is complex. This relationship is highly influenced by the anatomical site of injection and the specific characteristics of the injector’s technique. As previously demonstrated, reducing the applied and constant plunger rod speed leads to lower extrusion forces during automated in vitro injections [1]. This was confirmed herein and suggested that there is a nuanced interplay between the physical properties of the injected material, the technique used, and the anatomical site of injection (Table 6). Additionally, in comparison with automated setups, clinicians pause (i.e., while applying a steady force) during the injection process, to reposition the needle. In more detail, the mathematical formula that describes the depth of HA gel injections is based on the sinus of the needle penetration angle and the length of the needle (i.e., as implanted in the skin), which further illustrates the precision required in performing these procedures [5]. This precision level, coupled with the variability in skin thickness across different anatomical sites, underscores the challenges in standardizing injection techniques.

Given the diversity in injector sensitivity and technique (e.g., choice of needle or cannula, point-by-point, retro-tracing, or bolus methods), there is a significant variation in how each injector applies force. This variability is compounded by the inherent differences in skin anatomy, where the thickness of the epidermis, dermis, and hypodermis varies significantly across different body regions [5,8]. Moreover, ultrasound and MRI examinations align with histological data in revealing the layered complexity of the skin, challenging the notion of a uniform approach to injections [5,8]. The fact that some specialists identify only two layers within the dermis, likely due to limitations in imaging sensitivity, further complicates the endeavour to standardize injection techniques.

Overall, it appears clearly that variations in dermal filler injection speed, geometry, and applied force play a critical role in clinical practice, affecting the efficacy and outcomes of HA-based hydrogel injections. Notably, the individualized nature of the retained injection techniques, influenced by an injector’s aesthetic perspective and delicacy in gesture, makes it difficult to uniformize practices for treating wrinkles or volume loss. This variability, alongside the anatomical and histological diversity of the skin, calls for further research, including multicentric studies involving injectors from various medical specializations. Such studies would potentially provide deeper insights into the optimal applications of specific injection techniques, accommodating the broad range of human anatomy and injector preferences.

### 2.8. Influence of Tailored Clinical Protocols on Dermal Filler Injection Force Profiles

Generally, the impact of the retained injection technique on the resulting injection force profile is significant, as highlighted by the variation in resistance encountered when injecting into the dermis as compared to the hypodermis (Tables S1–S5). The dermis, composed of elastin and collagen fibres, offers more resistance to HA gel flow than the less dense hypodermic fat tissue [1,5]. Techniques such as bolus injection require higher pressures for a larger gel volume, contrasting with the more delicate point-by-point technique used primarily in the dermis [1]. This variability is further complicated by factors such as the injector’s visibility during the procedure and the speed of injection, which affect the extrusion force profile, especially in simulated skin environments [1]. Therefore, addressing the challenge of standardizing injection protocols is complex due to the inherent diversity in clinical injector sensitivity and preferences. Therein, some practitioners favour sharp needles, while others prefer cannulas, and similarly, there is high diversity in the adoption of point-by-point versus retro-tracing or bolus techniques across different layers of the skin. Importantly, this individualized or tailored approach to dermal filler injections reflects a broader resistance to clinical protocol standardization that could potentially homogenize aesthetic outcomes, erasing the unique characteristics that define our diverse global identities. The concern over a “one-size-fits-all” approach extends to cultural and aesthetic diversity, underscoring the importance of maintaining individuality in aesthetic treatments. 

Notwithstanding, the potential for standardizing dermal filler injection forces through technology, such as the electronic “gun”-like TeosyalPen^®^, is acknowledged. This device offers a more uniform injection pressure, which some patients may find less painful, yet it may not accommodate all injection techniques. This consideration highlights a potential avenue for reducing variability in injection outcomes without compromising the essential personalized approach in aesthetic medicine. Overall, while the different injection techniques and the anatomical site of injection significantly influence the force profiles of HA-based hydrogel injections, the practical feasibility of quantitatively standardizing injection protocols is limited by the diversity of practitioner techniques and the importance of maintaining individualized treatment approaches. Specifically, future strategies may include technological advancements that offer consistent injection pressures while still allowing for the nuanced, personalized techniques that reflect the unique identities and preferences of both practitioners and patients.

### 2.9. Study Limitations

The main technical limitations of the present study comprised the limited number of injectors and participants in the study. Furthermore, the number of in vivo MaiLi^®^ dermal filler injections was limited by the clinical treatment modalities, which did not enable the gathering of large datasets. As a result, the statistical comparison of the quantitative in vivo injectability data was not possible. As regards the low number of included patients, the study faced the challenge of finding individuals willing to be injected by two different professionals using different HA gels. Furthermore, the patients were made aware of the potential feeling of the FlexiForce^®^ sensor cable, which was not part of their usual treatment and could have occasioned minor discomfort. Moreover, the study’s methodology, particularly the exclusive use of sharp needles over cannulas, may not fully align with modern aesthetic practices, potentially biasing outcomes. The small sample size of five patients, due to the challenge of obtaining consent without financial incentives in Switzerland, severely limited the study’s generalizability. Furthermore, the non-multicentric nature of the study and the financial constraints restricted the scope and applicability of the findings to a broader context. Despite the clinical credibility added by the diverse backgrounds of the injectors, such elements introduced variability that could have influenced the results. Overall, the outlined limitations (i.e., methodological choices, small cohort, singular geographic focus, and financial restrictions) significantly impacted the findings’ generalizability. Therefore, future research should aim for larger, multicentric studies incorporating diverse injection techniques, HA gels, and injector specializations to enhance validity and applicability across the aesthetic medicine field.

As regards the low number of clinical injectors, the study included qualified and experienced practitioners as a counterbalancing measure, albeit two of them were new users of the MaiLi^®^ product range. Therefore, the discrepancy in specific clinical experience with the MaiLi^®^ product range could potentially have introduced some bias in the quantitative injectability measurements. Notwithstanding, all the injectors involved in this study had over a decade of experience in various injection techniques, administering treatments to approximately 10 patients daily in their respective practices. This extensive experience encompassed the different injection methods discussed in this paper, mirroring the expertise shared in our previous work examining 28 gels [1]. In that study, as in the current one, not all injectors utilized every gel, but they were all proficient in the four injection techniques evaluated both in vitro (i.e., using SimSkin^®^) and in vivo for injectors PM and TB. Specifically, PM has been using the entire range of the previously described 28 gels and the MaiLi^®^ brand since their introduction on the Swiss and European markets, even before FDA approval. Thus, while injectors TB and DP were not initially familiar with the MaiLi^®^ brand, it is important to highlight that all injectors possessed significant expertise in the required injection techniques. This foundational skillset ensured a level of consistency and proficiency across all treatments, mitigating potential biases related to unfamiliarity with specific products. Moreover, the diverse experiences and practices among the included injectors enriched the study’s findings, providing a comprehensive understanding of MaiLi^®^ product performance under real-world conditions. The injectors’ long-standing experience and adaptability to different HA gel technologies, including the adoption of new products like MaiLi^®^, further diminished the likelihood of technique bias or skewed measurement/outcome evaluations. 

Another limitation of the study consisted of the basic experimental biophysical attribute characterization for the considered hydrogels (i.e., rheology), as the applied methodologies constitute common debate elements [38,39,40]. Specifically, additional investigation into chemical and structural aspects of the cross-linked polymeric network of MaiLi^®^ products could potentially have enabled some form of elucidation of their optimal injectability attributes (i.e., as demonstrated herein). Specifically, it is well known that chemical modifications of the HA polymer network and the addition of functional excipients bear critical impacts on hydrogel system behaviour and efficacy [41,42,43,44,45]. Notwithstanding, it is also recognized that the manufacturing process parameters themselves (e.g., homogenization, sterilization) and their fine-tuning bear significant impacts on finished product attributes, thus endpoint analytical controls would probably prove insufficient to holistically assess any given manufacturing technology (e.g., OxiFree™) [1,46,47]. 

Finally, few comparisons of the reported original injectability data with literature sources were performed herein, as the study was mainly designed as a continuation of previous work by the authors [1]. Specifically, while similar trends and influencing factors were found to be conserved in the present study (i.e., same operators and experimental setups), the obtained datasets confirmed that MaiLi^®^ dermal fillers clearly stand out in terms of in-use injectability behaviour [1]. Of note, the domain of cross-linked HA-based dermal filler injectability (i.e., especially in vivo) is poorly represented in the literature (which focuses primarily on system rheology), which constituted a main rationale element for the design of the present study [48,49,50].

### 2.10. Future Perspectives

Specific future perspectives to the present study comprise the use of larger injector and participant pools in order to further augment the robustness of the obtained datasets. Specifically, larger and multicentric investigations by injectors of diverse clinical experience levels would add to the translational relevance of the study, with in vivo data gathered under real-world conditions. Secondly, the use of micro-canulas instead of the provided needles would be of high interest for MaiLi^®^ dermal filler product injectability assessments, as such administration devices are often used in modern clinical practice. Thirdly, it would be of high interest to prospectively investigate if the reported qualitatively enhanced injectability levels of MaiLi^®^ dermal fillers correlate with an enhanced care experience by the patients and/or the practitioners. 

Furthermore, future research should focus on quantifying the optimum force profile required for even and smooth dermal filler extrusion. This research would not only deepen the understanding of HA gel properties but also ameliorate clinical practices by guiding physicians in achieving better, more consistent aesthetic results. Finally, future directions of research could comprise the use of advanced imaging techniques like ultrasound and MRI to observe the behaviour of HA gels in vivo, providing non-invasive and detailed insights into how these gels perform within the skin and hypodermis over time. Addressing these points could tangibly bridge the gap between bench research and bedside application, enhancing the utility of HA gels in aesthetic medicine.

## 3. Conclusions

The present study provided comprehensive injectability datasets on four widely used MaiLi^®^ dermal filler variants. The study was designed to build on previous reports of cross-linked HA dermal filler injectability attribute assessments, with enhanced translational and clinical relevance. While quantitative differences were observed between the automated and manual injectability setups (i.e., in vitro and in vivo), highly consistent qualitative aspects of filler injectability were set forth for the MaiLi^®^ product variants. Specifically, it was shown that very smooth injection force plateaus could be obtained for all four MaiLi^®^ variants and that the respective mean plateau forces were not statistically different. 

Overall, the experimental results confirmed a high degree of hydrogel system homogeneity in terms of viscoelasticity and injectability attributes, which is most notable for a whole range of products with varying quantitative compositions. The observational case reports for in vivo product injectability evaluation underscored the importance of injection speed and the area of the thumb used for the administration. The main conclusion of the study was that MaiLi^®^ products present highly conserved qualitative and quantitative injectability attributes across multiple variants, which is distinctive in the field. Overall, the presented work underscored the central importance of thoroughly and systematically examining product attributes from a clinically relevant quality viewpoint, in order to rationally select the best available options for medical aesthetic care. 

## 4. Materials and Methods

### 4.1. Reagents and Consumables Used in the Study

Physiological saline solution (NaCl 0.9%) was purchased from Bichsel (Unterseen, Switzerland). A total of four different cross-linked HA-based commercial dermal fillers from the MaiLi^®^ brand were purchased from the product manufacturer (Sinclair Pharma Ltd., London, UK). The investigated MaiLi^®^ Precise lots were N°211322-1, N°222456-2, N°211322-2, and N°213561-1. The investigated MaiLi^®^ Define lots were N°202775-1, N°223419-2, and N°212916-1. The investigated MaiLi^®^ Volume lots were N°202775-2, N°211228-2, and N°211803-2. The investigated MaiLi^®^ Extreme lots were N°202843-1, N°222773-1, N°211678-2, and N°213561-2. The various needles used in the study were taken directly from each corresponding product packaging and comprised 30 G × ½″ needles (0.30 × 13 mm; TSK Laboratories, Tochigi-Ken, Japan) and 27 G × ½″ needles (0.40 × 13 mm; TSK Laboratories, Tochigi-Ken, Japan). For the technical needs of the study, TEOSYAL RHA^®^ 2 products were purchased from TEOXANE (Geneva, Switzerland). For establishing standardized in vitro dermal filler injectability assessment conditions, synthetic SimSkin^®^ cutaneous equivalents were purchased from Wallcur (San Diego, CA, USA). 

### 4.2. MaiLi^®^ Dermal Filler Product Rheological Characterization

The basic rheological behaviours of the four investigated MaiLi^®^ product variants were determined in oscillatory rheology. Measurements were performed on an HR 10 rheometer (TA Instruments, Guyancourt, France) equipped with a Peltier plate–plate measuring geometry. Each measurement was performed in triplicate on 700 µL of undiluted hydrogel sample. The complex viscosity (η*) values of the samples were determined as a function of the applied oscillatory frequency. To this end, a frequency sweep was performed from 0.1 Hz to 10 Hz at a fixed temperature of 25 °C. The constant shear stress was set at 2 N·m^−2^ in all experiments.

### 4.3. MaiLi^®^ Dermal Filler Comparative Manual Injectability Studies in SimSkin^®^ Cutaneous Equivalents

For the in vitro comparative assessments of MaiLi^®^ dermal filler product injectability attributes, a SimSkin^®^ cutaneous equivalent model was used. SimSkin^®^ units consist of polymeric epidermis, dermis, and subcutaneous layers. The total substrate thickness is of 0.6 cm (i.e., 0.3 cm for the epidermis, 0.2 cm for the dermis, and 0.1 cm for the subcutaneous layer) [1]. Quantitative injectability measurements were performed by three experienced clinicians, using a dynamometric sensor (FlexiForce^®^ Quickstart Board, Tekscan, Norwood, MA, USA) connected to myDAQ for data acquisition (National Instruments, Austin, TX, USA). For the assays, the plunger rods of the four MaiLi^®^ product variants were replaced with those of TEOSYAL RHA^®^ 2 products, which were equipped with the hilt-mounted sensor, as previously described [1]. 

The injection force parameters of the four MaiLi^®^ dermal fillers were all determined using the original syringes and needles provided by the manufacturer. The mean forces of injection and peak forces of injection were automatically recorded. For each injection, the needle was introduced tangentially to the skin plane, at an angle of <10° for intradermal injections and of <19° for hypodermal injections. For point-by-point injections, only the bevel of the needle was introduced into the skin and small quantities of hydrogel were placed at regular intervals. For retro-tracing injections, the whole needle body was introduced into the skin and was pulled out simultaneously with the hydrogel injection.

For each MaiLi^®^ product variant, four types of hydrogel product administration modalities (i.e., both indicated uses and off-label uses) were retained and were performed in triplicate by each injector. Namely, intradermal point-by-point and retro-tracing injections were performed, followed by hypodermal bolus and retro-tracing injections in SimSkin^®^ substrates. Based on the routine clinical practices of each injector, the thumb pulp or the P1-P2 thumb joint was used to manually and sequentially inject the investigated products. Specifically, the methods of injection closely mirrored those routinely used in the clinical practices of the co-authors for facial wrinkle filling or for volumetric supplementation. Of note, only injector PM was clinically experienced with the MaiLi^®^ product range, whereas injectors TB and DP had not previously used the investigated products [17].

### 4.4. Comparative Automated Injectability Studies of MaiLi^®^ Dermal Fillers in SimSkin^®^ Cutaneous Equivalents at Constant Injection Speeds

To complement the manual injectability experiments, automated injectability assessments were performed in a dedicated in vitro texture analysis setup. The injection force profiles of the four commercially available MaiLi^®^ product variants were determined in triplicate using the original syringes and needles supplied by the manufacturer. For the assays, the plunger rods of the four MaiLi^®^ product variants were replaced with those of TEOSYAL RHA^®^ 2 products, as previously described [1]. The hydrogel products were injected into SimSkin^®^ cutaneous equivalents by a Texture Analyzer TA.XT. Plus instrument (Tracomme, Schlieren, Switzerland). The needle body was inserted perpendicularly to the skin plane at the appropriate depth within the synthetic scaffold for hydrogel injection. Constant plunger rod actuation speeds of 0.2 mm·s^−1^ and 1 mm·s^−1^ were used at ambient temperature (i.e., 25 °C). The higher injection speed value of 1 mm·s^−1^ was used for preliminary experiments in order to best discriminate the products in terms of hydrogel system intra-syringe homogeneity. The lower injection speed value of 0.2 mm·s^−1^ was then used to better approximate clinical manual dermal filler product injection conditions. The analysis of the injection force profiles enabled us to calculate the mean plateau injection forces for each MaiLi^®^ product variant. 

### 4.5. Clinical Case Reports on the In Vivo Injectability Attributes of MaiLi^®^ Dermal Fillers

For the obtention of in vivo injectability data for MaiLi^®^ dermal fillers, a series of five case reports was gathered from the routine clinical practice of co-author PM. Therefore, five female participants were included in this study, following oral and written information and a fixed consideration period of fifteen days. This study was compliant with the declaration of Helsinki and the applicable Swiss laws on copyright [51,52]. Four female participants were of Caucasian ethnicity and one female participant was of Hispanic ethnicity. The mean participant age was 62 years. All participants explicitly consented to the gathering of in vivo injection force data using the FlexiForce^®^ dynamometric sensor during their routine clinical treatment. In parallel and subsequently to the in vivo injections, the same MaiLi^®^ dermal filler administration regimens were carried out with SimSkin^®^ cutaneous equivalents. Generally, the product administration methodology employed for the in vivo part of the presented study was consistent with that of previous reports by the authors [5].

#### 4.5.1. In Vivo Intradermal Point-by-Point Injection Methodology

For intradermal point-by-point injections, the needle bevel (i.e., approximately 1 mm in length) was introduced under the skin at an angle of <10°, reaching the medium dermis. Plunger rod actuation then allowed to dispense a small quantity of hydrogel in order to slightly raise the skin surface into an artificial papule. The needle bevel was then introduced under the skin again, following the wrinkle line and adjacent to the first papule, before the injection process was repeated. This sequence was performed iteratively until the whole wrinkle was assessed as being filled. 

#### 4.5.2. In Vivo Intradermal Retro-Tracing Injection Methodology

For intradermal retro-tracing injections, the whole needle body was introduced under the skin at an angle of <10°, reaching the medium dermis. Plunger rod actuation was then performed simultaneously with the slow backwards pulling out of the needle from the skin. This sequence was performed iteratively until the whole wrinkle was assessed as being filled. 

#### 4.5.3. In Vivo Intradermal Antero-Tracing Injection Methodology

This type of injection was initiated by placing the needle bevel in the same position as for the point-by-point injections. Then, the whole needle body was progressively introduced further into the skin (i.e., in a trajectory parallel to the skin surface), where simultaneous and constant plunger rod actuation enabled to gently dispense the hydrogel. This sequence was performed iteratively until the whole wrinkle was assessed as being filled. 

#### 4.5.4. In Vivo Hypodermal Bolus Injection Methodology

For hypodermal bolus injections, the needle bevel was placed in the hypodermis, at a perpendicular angle with the skin plane (i.e., to reach the distal close-to-the-bone layer) or at an angle of <19° with the skin plane (i.e., to reach the fat layer). Continuous plunger rod actuation then enabled to deposit a relatively large amount of hydrogel at a constant depth.

#### 4.5.5. In Vivo Hypodermal Retro-Tracing Injection Methodology

This type of injection was performed in the same way as the intradermal retro-tracing injection but using an angle of <19° between the needle and the skin plane. 

### 4.6. Statistical Analysis and Data Presentation

Data were reported as mean values accompanied by the corresponding standard deviations as error bars, wherever applicable. For the statistical comparison of values from multi-group quantitative datasets, a one-way ANOVA or a two-way ANOVA test was performed and was followed by a post hoc Tukey’s multiple comparison test. A *p*-value < 0.05 was retained as a general base for statistical significance determination. Detailed levels of statistical significance can be found in the Section 2. The statistical calculations and/or data presentation were performed using Microsoft Excel (Microsoft Corporation, Redmond, WA, USA), Microsoft PowerPoint, and GraphPad Prism v. 8.0.2 (GraphPad Software, San Diego, CA, USA).

## Figures and Tables

**Table 1:** Comparative overview of the technical specifics of the MaiLi^®^ dermal filler product variants included in the study. The data were compiled from manufacturer-provided sources. G, gauge; HA, hyaluronic acid; N/A, non-applicable.

Product Variant	Clinical Uses ^1^	Needle Size (G × Length) ^2^	HA Concentration (mg/mL)	Cross-Linked HA (Y/N)	Lidocaine Presence (Y/N) ^3^	HA Cross-Linking Technology
Precise	Fine lines	30 G × ½″	15.0 mg/mL	Yes	Yes	OxiFree™
Define	Medium folds; lip volume	30 G × ½″	18.0 mg/mL	Yes	Yes	OxiFree™
Volume	Volumizer	27 G × ½″	21.0 mg/mL	Yes	Yes	OxiFree™
Extreme	Cheeks; temples; jaw line; chin volumizer	27 G × ½″	24.0 mg/mL	Yes	Yes	OxiFree™
NaCl Control	N/A	30 G × ½″	0.0 mg/mL	N/A	No	N/A

^1^ Product clinical uses, as specified by the manufacturer [22]. ^2^ The original needles, as supplied with the MaiLi^®^ product syringes, are manufactured by TSK, Tochigi-Ken, Japan. ^3^ Lidocaine contents are consistent across the whole MaiLi^®^ dermal filler product range, with 0.3% *m*/*v* lidocaine.

**Table 2:** Quantitative data on the manual injection forces required to inject the MaiLi^®^ Precise dermal filler in SimSkin^®^ cutaneous equivalents. Experiments were performed by three qualified operators using various injection techniques and in vitro administration sites. DP, Daniel Perrenoud; N, Newtons; PM, Patrick Micheels; TB, Thierry Bezzola.

Injection Parameters		Injection Force in the Dermis (N)		Injection Force in the Hypodermis (N) ^1^		Mean Values/Injector (N)
		Point-by-Point	Retro-Tracing	Bolus	Retro-Tracing	
Injectors	PM	0.46	0.36	0.54	0.73	0.52 ± 0.16
	TB	2.01	1.60	0.83	1.65	1.52 ± 0.50
	DP	0.33	0.52	0.48	0.53	0.46 ± 0.09
Mean Values/Injection Site (N)		0.93 ± 0.93	0.83 ± 0.67	0.62 ± 0.19	0.97 ± 0.60	Global Mean Value (N) 0.84 ± 0.58

^1^ Hypodermal injections are not part of the specified indications for use of this MaiLi^®^ product variant (Table 1).

**Table 3:** Quantitative data on the manual injection forces required to inject the MaiLi^®^ Define dermal filler in SimSkin^®^ cutaneous equivalents. Experiments were performed by three qualified operators using various injection techniques and in vitro administration sites. DP, Daniel Perrenoud; N, Newtons; PM, Patrick Micheels; TB, Thierry Bezzola.

Injection Parameters		Injection Force in the Dermis (N)		Injection Force in the Hypodermis (N) ^1^		Mean Values/Injector (N)
		Point-by-Point	Retro-Tracing	Bolus	Retro-Tracing	
Injectors	PM	0.82	1.04	1.28	1.77	1.23 ± 0.41
	TB	1.15	1.13	1.42	1.25	1.24 ± 0.13
	DP	0.77	0.91	0.87	0.75	0.83 ± 0.08
Mean Values/Injection Site (N)		0.91 ± 0.21	1.02 ± 0.11	1.19 ± 0.29	1.26 ± 0.51	Global Mean Value (N) 1.10 ± 0.30

^1^ Hypodermal injections are not part of the specified indications for use of this MaiLi^®^ product variant (Table 1).

**Table 4:** Quantitative data on the manual injection forces required to inject the MaiLi^®^ Volume dermal filler in SimSkin^®^ cutaneous equivalents. Experiments were performed by three qualified operators using various injection techniques and in vitro administration sites. DP, Daniel Perrenoud; N, Newtons; PM, Patrick Micheels; TB, Thierry Bezzola.

Injection Parameters		Injection Force in the Dermis (N) ^1^		Injection Force in the Hypodermis (N)		Mean Values/Injector (N)
		Point-by-Point	Retro-Tracing	Bolus	Retro-Tracing	
Injectors	PM	1.84	1.75	1.91	2.28	1.95 ± 0.23
	TB	0.81	0.79	0.91	0.85	0.84 ± 0.05
	DP	0.51	0.45	0.51	0.44	0.48 ± 0.04
Mean Values/Injection Site (N)		1.05 ± 0.70	1.00 ± 0.67	1.11 ± 0.72	1.19 ± 0.97	Global Mean Value (N) 1.09 ± 0.66

^1^ Intradermal injections are not part of the specified indications for use of this MaiLi^®^ product variant (Table 1).

**Table 5:** Quantitative data on the manual injection forces required to inject the MaiLi^®^ Extreme dermal filler in SimSkin^®^ cutaneous equivalents. Experiments were performed by three qualified operators using various injection techniques and in vitro administration sites. DP, Daniel Perrenoud; N, Newtons; PM, Patrick Micheels; TB, Thierry Bezzola.

Injection Parameters		Injection Force in the Dermis (N) ^1^		Injection Force in the Hypodermis (N)		Mean Values/Injector (N)
		Point-by-Point	Retro-Tracing	Bolus	Retro-Tracing	
Injectors	PM	0.58	0.35	0.60	0.54	0.52 ± 0.13
	TB	0.96	1.02	1.32	1.35	1.16 ± 0.20
	DP	0.46	0.41	0.51	0.70	0.52 ± 0.13
Mean Values/Injection Site (N)		0.67 ± 0.26	0.59 ± 0.37	0.81 ± 0.44	0.86 ± 0.43	Global Mean Value (N) 0.73 ± 0.35

^1^ Intradermal injections are not part of the specified indications for use of this MaiLi^®^ product variant (Table 1).

**Table 6:** Quantitative data of plateau injection force ^1^ determination in automated in vitro injectability assays at a constant plunger rod actuation speed of 0.2 mm·s^−1^ and 1 mm·s^−1^ for the four MaiLi^®^ product variants. No statistical difference (i.e., *p*-value > 0.05) was found as regards the inter-variant mean plateau force of injection at a given speed. Extremely significant statistical differences (i.e., *p*-value < 0.0001) were found between all values when comparing the respective results at different injection speeds. N, Newtons.

Product Variant	Low Injection Speed (0.2 mm·s^−1^)			High Injection Speed (1 mm·s^−1^)		
	Plateau Minimum Force (N)	Plateau Maximum Force (N)	Plateau Mean Force (N)	Plateau Minimum Force (N)	Plateau Maximum Force (N)	Plateau Mean Force (N)
MaiLi^®^ Precise	10.2 ± 0.3	11.8 ± 0.2	11.2 ± 0.5	32.7 ± 0.4	37.4 ± 0.6	35.8 ± 1.3
MaiLi^®^ Define	10.0 ± 0.4	11.3 ± 0.3	10.1 ± 1.1	34.8 ± 1.9	40.5 ± 0.6	36.4 ± 2.7
MaiLi^®^ Volume	9.2 ± 0.5	10.8 ± 0.2	10.6 ± 0.6	33.6 ± 1.3	36.4 ± 2.3	34.9 ± 1.9
MaiLi^®^ Extreme	9.8 ± 0.4	10.8 ± 0.4	10.0 ± 0.9	35.0 ± 1.1	39.5 ± 1.2	36.8 ± 1.1
NaCl Control	3.8 ± 0.1	4.3 ± 0.2	4.0 ± 0.2	3.9 ± 0.1	4.2 ± 0.1	4.0 ± 0.1

^1^ Plateau force values were determined between 40% and 95% of the plunger travel distance during a full hydrogel unit extrusion cycle.

**Table 7:** Summary of the anonymized demographic and treatment-related data for the in vivo portion of the study. The five included female participants were all treated with MaiLi^®^ dermal filler variants during routine maintenance visits. G, Gauge.

Participant N° (Age)	Fitzpatrick/Glogau Class	Treated Areas (Injection Techniques)	MaiLi^®^ Product Variant	Needle Size (G) ^1^	Injection Depth
1 (64)	II/III	Galbella (Point-by-point); Nasolabial folds (Antero-tracing, Retro-tracing); Marionet (Point-by-point)	Precise	30 G × ½″	Mid-reticular dermis
2 (54)	II/III	Glabella (Point-by-point); Nasolabial folds (Point-by-point); Marionet (Retro-tracing)	Define	30 G × ½″	Mid-reticular dermis
3 (75)	III/III	Cheeks (Bolus); Nasolabial folds (Retro-tracing, Point-by-point); Marionet (Retro-tracing, Point-by-point)	Volume	27 G × ½″	Fat; deep dermis/hypodermis; deep dermis/hypodermis
4 (64)	IV (hispanic)/III	Cheeks (Bolus)	Extreme	27 G × ½″	Close-to-the-bone
5 (53)	III/III	Cheeks (Bolus)	Extreme	27 G × ½″	Fat

^1^ All MaiLi^®^ dermal filler injections were performed with needles and no cannulas were used in the study.

## Data Availability

The data presented in this study are openly available within the article files.

## Supplementary Materials

The following supporting information can be downloaded at https://www.mdpi.com/article/10.3390/gels10040276/s1, Figure S1: Illustrations of the SimSkin^®^ cutaneous equivalent and its uses; Figure S2: In vitro injection force profiles for the MaiLi^®^ Precise dermal filler; Figure S3: In vitro injection force profiles for the MaiLi^®^ Define dermal filler; Figure S4: In vitro injection force profiles for the MaiLi^®^ Volume dermal filler; Figure S5: In vitro injection force profiles for the MaiLi^®^ Extreme dermal filler; Figure S6: Illustration of dermal filler injection techniques; Figure S7: In vivo dermal filler injection force profiles; Figure S8: In vitro and in vivo dermal filler injection force profiles; Figure S9: In vitro and in vivo dermal filler injection force profiles; Figure S10: In vitro and in vivo dermal filler injection force profiles; Table S1: In vivo injectability data for the MaiLi^®^ Precise dermal filler; Table S2: In vivo injectability data for the MaiLi^®^ Define dermal filler; Table S3: In vivo injectability data for the MaiLi^®^ Volume dermal filler; Table S4: In vivo injectability data for the MaiLi^®^ Extreme dermal filler; Table S5: In vivo injectability data for the MaiLi^®^ Extreme dermal filler.

## Abbreviation List

BDDE	1,4-butanediol diglycidyl ether
CE	European mark of conformity
DP	Daniel Perrenoud
η*	complex viscosity
G	gauge
HA	hyaluronic acid
Hz	Hertz
MRI	magnetic resonance imaging
N	Newtons
N/A	non-applicable
Pa·s	Pascal seconds
PM	Patrick Micheels
TB	Thierry Bezzola
UK	United Kingdom
USA	United States of America
