# Supplementary material for: Comprehensive Evaluation of Injectability Attributes in OxiFree™ Dermal Fillers: MaiLi® Product Variants and Clinical Case Reports
Source: Gels. 2024 Apr 19;10(4):276. doi: 10.3390/gels10040276 (PMC11049332; doi:10.3390/gels10040276)
Supplement: Supplementary file 1 [file gels-10-00276-s001.zip › Gels-2946321 Supplementary Materials.pdf]

gels

Supplementary Materials:

Comprehensive Evaluation of Injectability Attributes in OxiFree™ Dermal Fillers: MaiLi® Product Variants and Clinical Case Reports

Patrick Micheels *, Alexandre Porcello, Thierry Bezzola, Daniel Perrenoud, Marie-Odile Christen, Lee Ann Applegate and Alexis Laurent *

1. Supplementary Figures

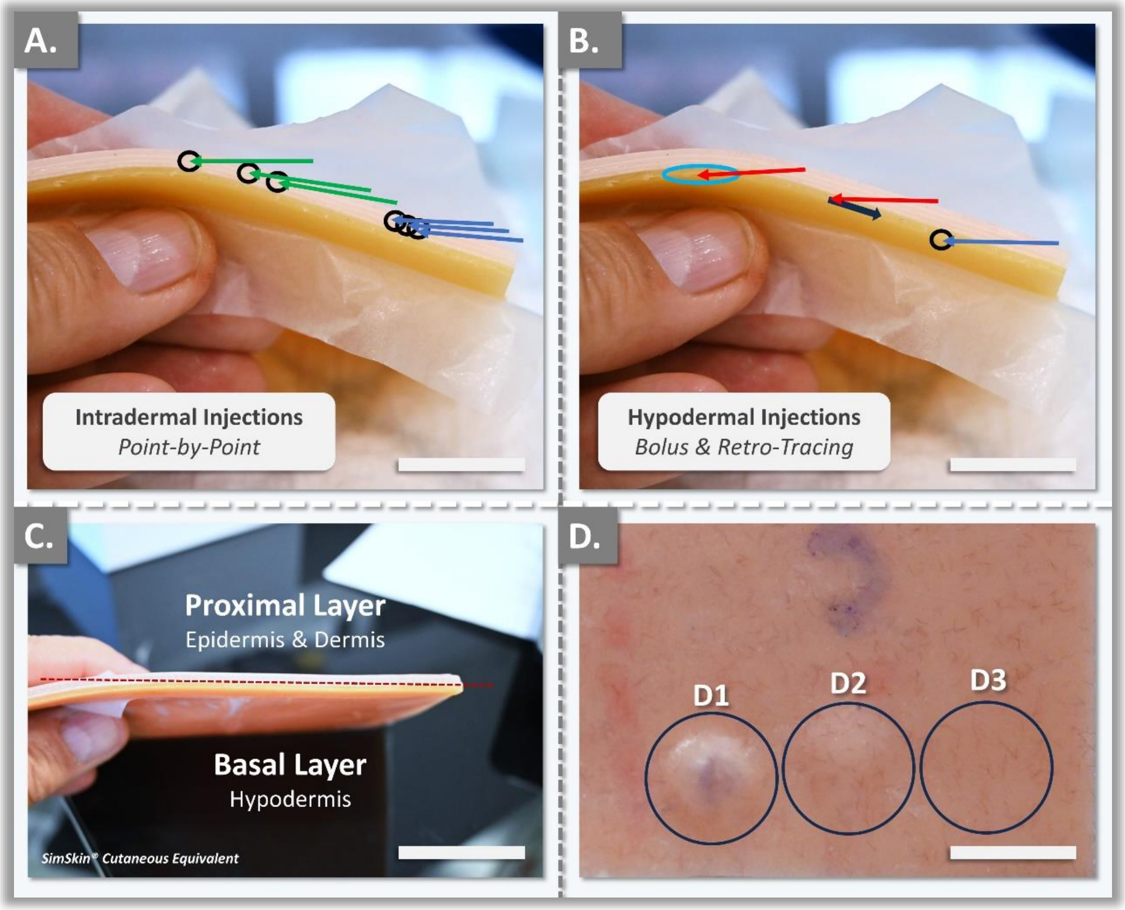

Figure S1. Photographic illustration of the SimSkin® cutaneous equivalent, used for the standardized in vitro injectability assessments of the MaiLi® cross-linked HA-based dermal fillers. (**A**) Illustration of the administration sites for intradermal point-by-point injections. The needle (e.g., green and blue arrows) is inserted at an angle of < 10° with the skin plane, for optimal control of the injection depth. For in vitro injections in SimSkin® substrates, the spacing represented by the green arrows was used, whereas the spacing represented by the blue arrows was used for patient clinical treatments. Scale bar = 10 mm. (**B**) Illustration of the administration sites (e.g., administration depth illustrated by the black circle) for hypodermal bolus and retro-tracing injections (i.e., light blue site, with the directionality illustrated by the black arrow). The needle is inserted at an angle of < 19° with the skin plane, for optimal control of the injection depth. Scale bar = 10 mm. (**C**) Annotated side view of the SimSkin® cutaneous equivalent. Scale bar = 20 mm. (**D**) Enlarged top view (i.e., proximal layer, epidermis surface) of the administration sites following dermal filler injection in the superficial dermis (i.e., point-by-point; **D1**), in the medium-depth dermis (i.e., point-by-point; **D2**), and in the hypodermis (i.e., bolus; **D3**). Scale bar = 4 mm. HA, hyaluronic acid.

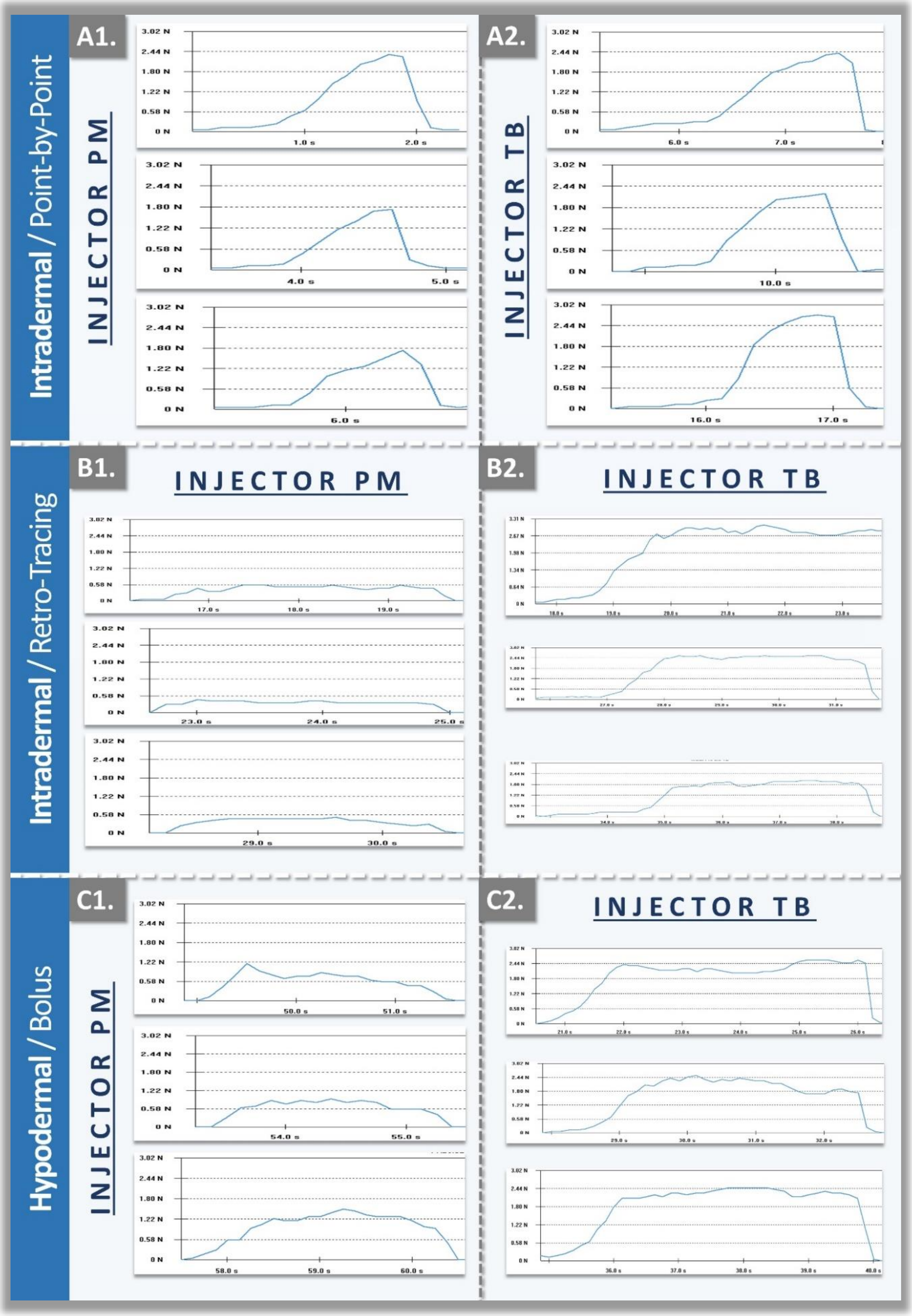

Figure S2. Experimental in vitro injection force profiles obtained manually for the MaiLi® Precise dermal filler in SimSkin® cutaneous equivalents. The injections were performed in triplicate by two clinicians. (**A1**,**A2**) Intradermal injections performed with the point-by-point injection technique. (**B1**,**B2**) Intradermal injections performed with the retro-tracing injection technique. (**C1**,**C2**) Hypodermal injections performed with the bolus injection technique. N, Newtons; PM, Patrick Micheels; TB, Thierry Bezzola.

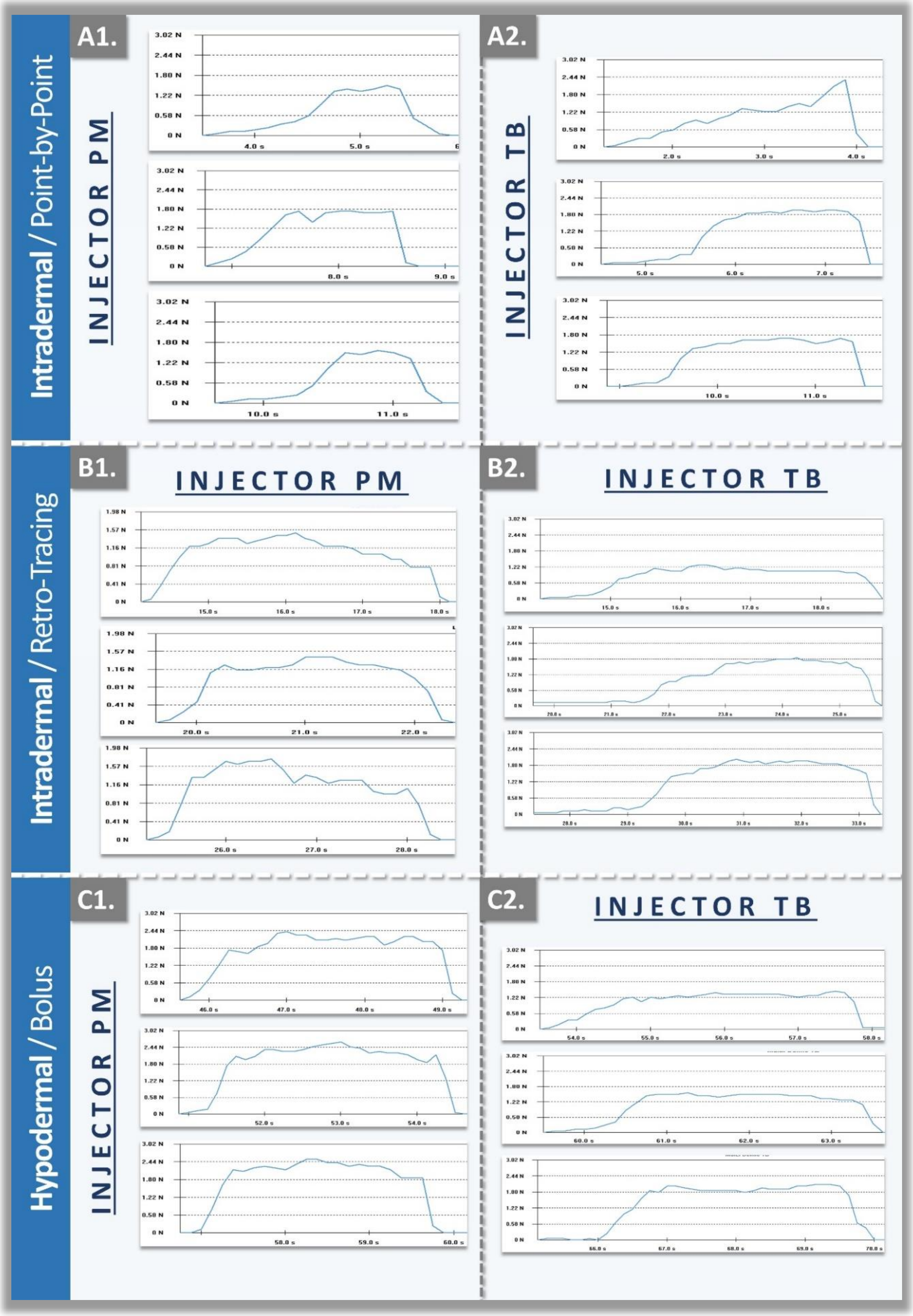

Figure S3. Experimental in vitro injection force profiles obtained manually for the MaiLi® Define dermal filler in SimSkin® cutaneous equivalents. The injections were performed in triplicate by two clinicians. (**A1**,**A2**) Intradermal injections performed with the point-by-point injection technique. (**B1**,**B2**) Intradermal injections performed with the retro-tracing injection technique. (**C1**,**C2**) Hypodermal injections performed with the bolus injection technique. N, Newtons; PM, Patrick Micheels; TB, Thierry Bezzola.

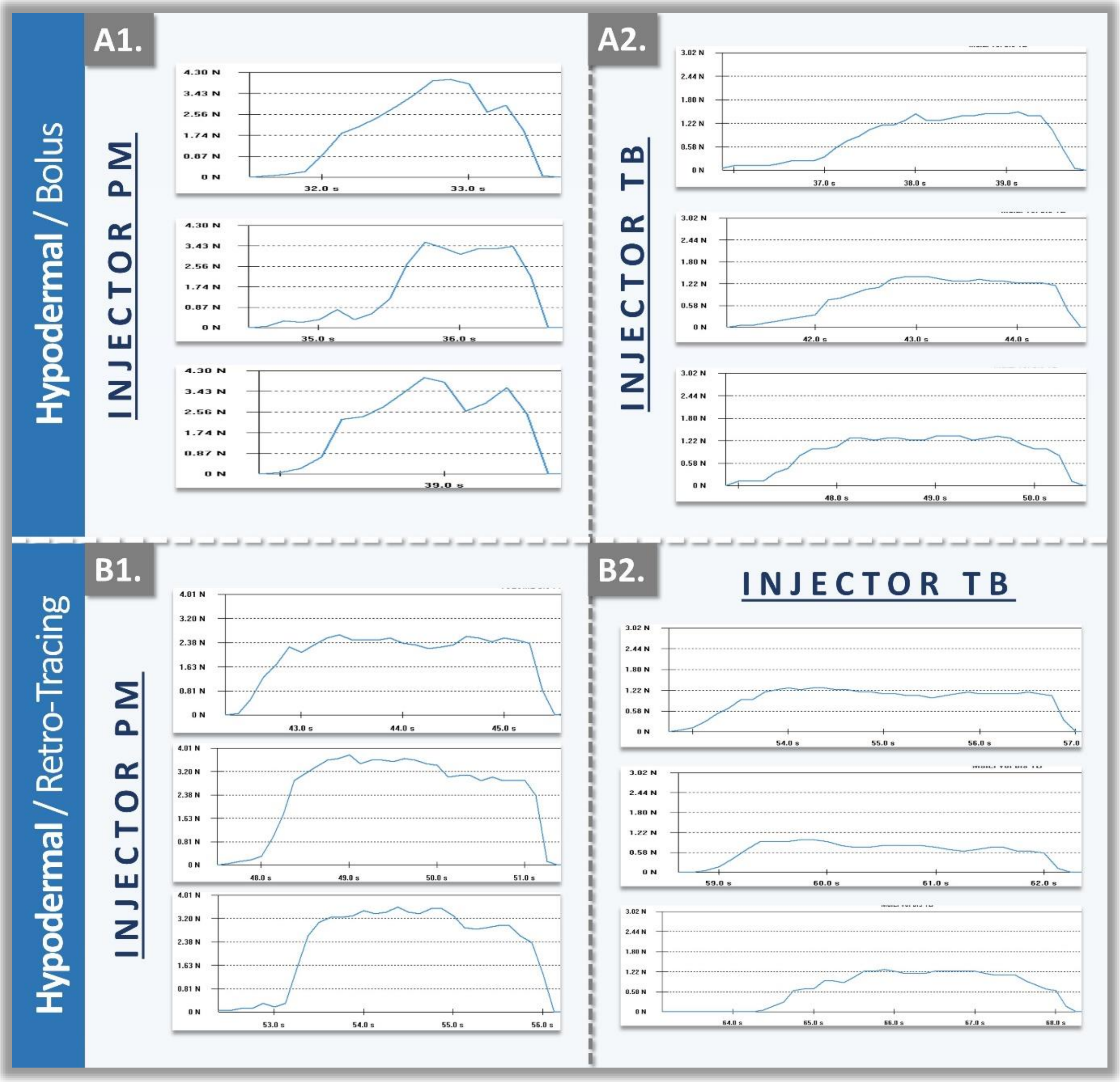

Figure S4. Experimental in vitro injection force profiles obtained manually for the MaiLi® Volume dermal filler in SimSkin® cutaneous equivalents. The injections were performed in triplicate by two clinicians. (**A1**,**A2**) Hypodermal injections performed with the bolus injection technique. (**B1**,**B2**) Hypodermal injections performed with the retro-tracing injection technique. N, Newtons; PM, Patrick Micheels; TB, Thierry Bezzola.

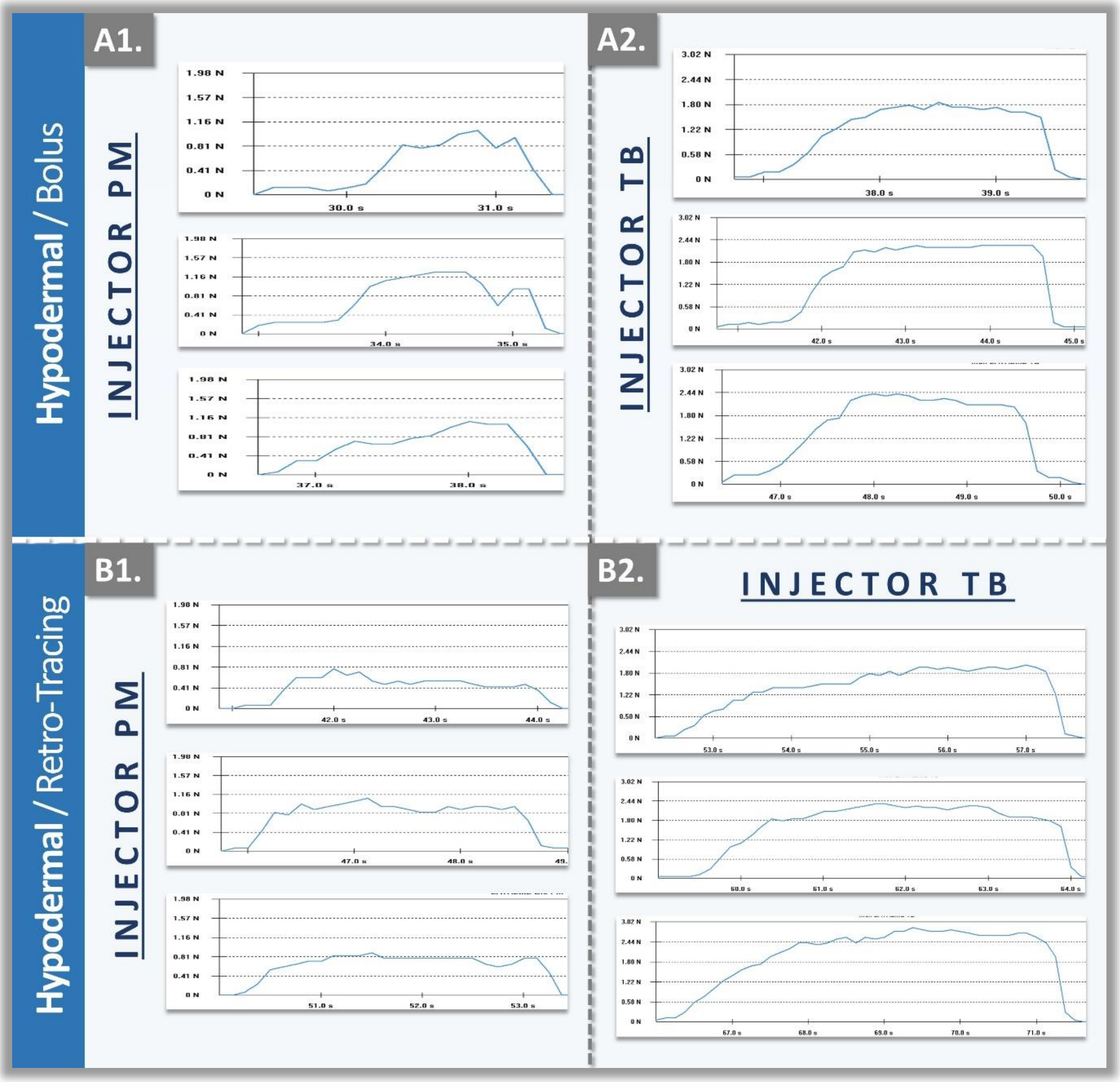

Figure S5. Experimental in vitro injection force profiles obtained manually for the MaiLi® Extreme dermal filler in SimSkin® cutaneous equivalents. The injections were performed in triplicate by two clinicians. (**A1**,**A2**) Hypodermal injections performed with the bolus injection technique. (**B1**,**B2**) Hypodermal injections performed with the retro-tracing injection technique. N, Newtons; PM, Patrick Micheels; TB, Thierry Bezzola.

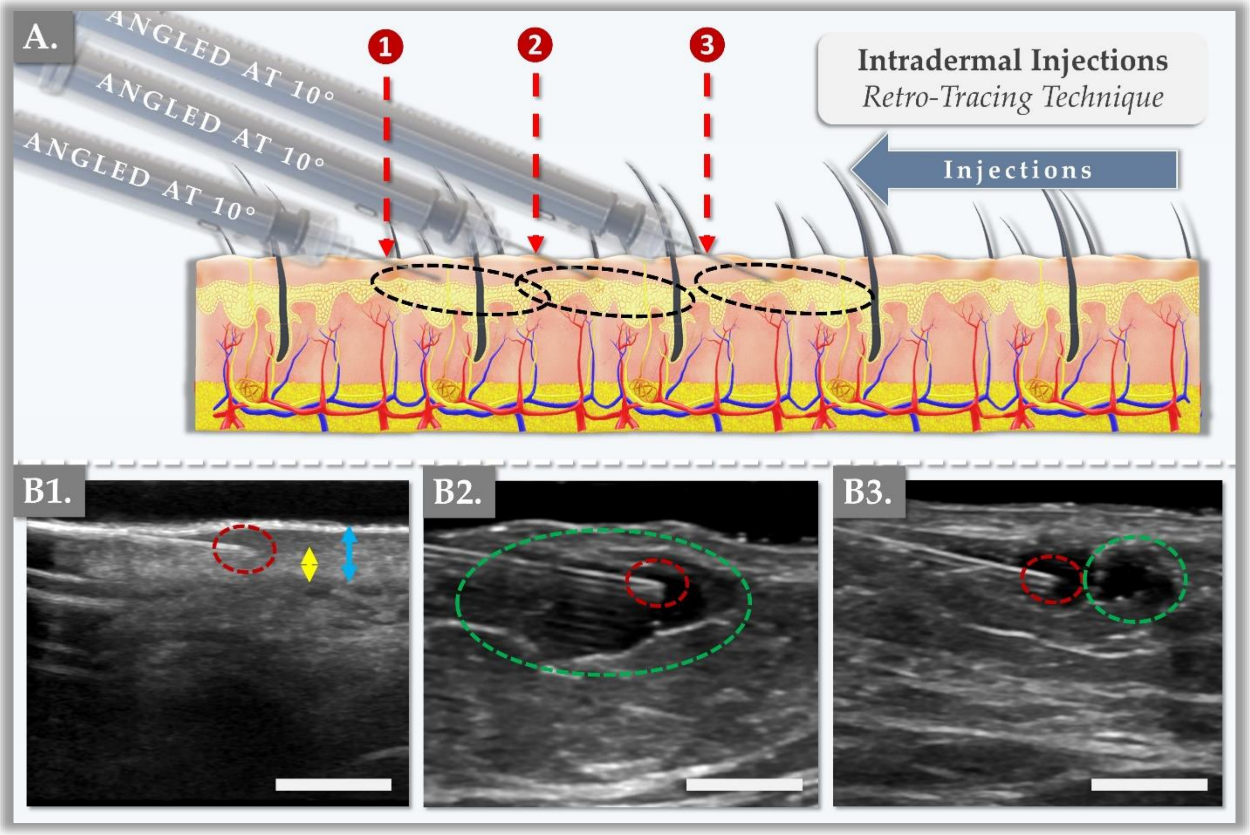

Figure S6. Illustrated close-ups of dermal filler administration. (**A**) Schematic illustration of the retro-tracing injection technique. The direction of progression of the injections is identified by the grey arrow. The successive entry points of the needle in the skin are identified by the red arrows. Hydrogel deposits are identified by black outlines, as defined by the retained injection angle and injection depth. (**B1**) Ultrasound view of point-by-point injections with a 30 G × ½′′ needle implanted in the mid-reticular dermis. The blue spacer identifies total dermis thickness and the yellow spacer identifies the reticular dermis thickness. The needle bevel is outlined in red. Scale bar = 0.75 mm. (**B2**) Ultrasound view of a bolus injection with a 30 G × ½′′ needle implanted in the hypodermis. The needle bevel (i.e., facing down) is outlined in red. Hydrogel deposits in the fat are outlined in green. Scale bar = 0.50 mm. (**B3**) Ultrasound view of a retro-tracing injection with a 27 G needle implanted in the hypodermis. The needle bevel is outlined in red. Hydrogel deposits in the fat are outlined in green. Scale bar = 0.50 mm. Illustration elements provided by Dr. Patrick Micheels, private archives.

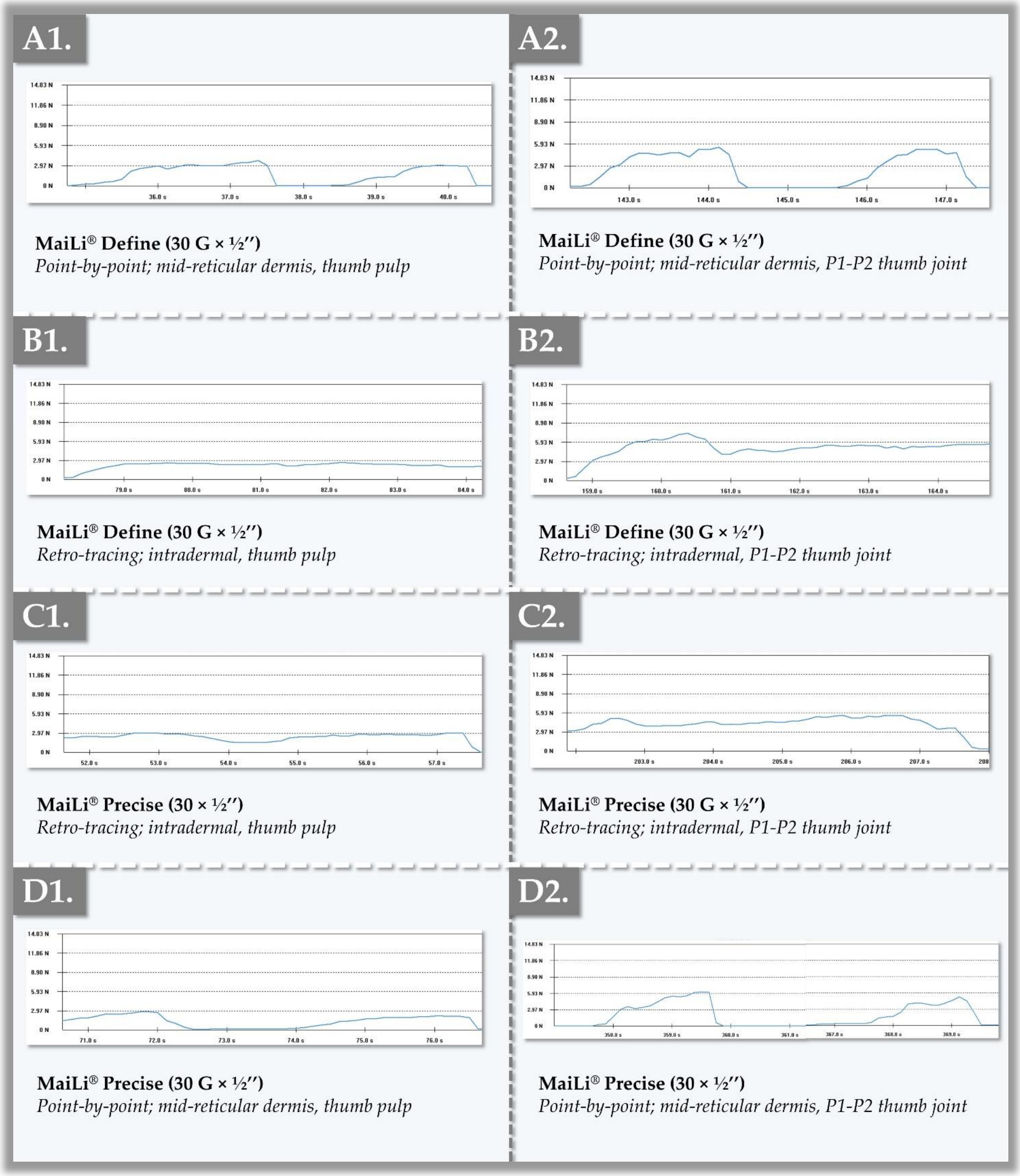

Figure S7. Injection force profiles of MaiLi® Define (**A1–B2**) and MaiLi® Precise (**C1–D2**) dermal fillers during in vivo injectability assessments (i.e., Participants N°1 and N°2). N, Newtons.

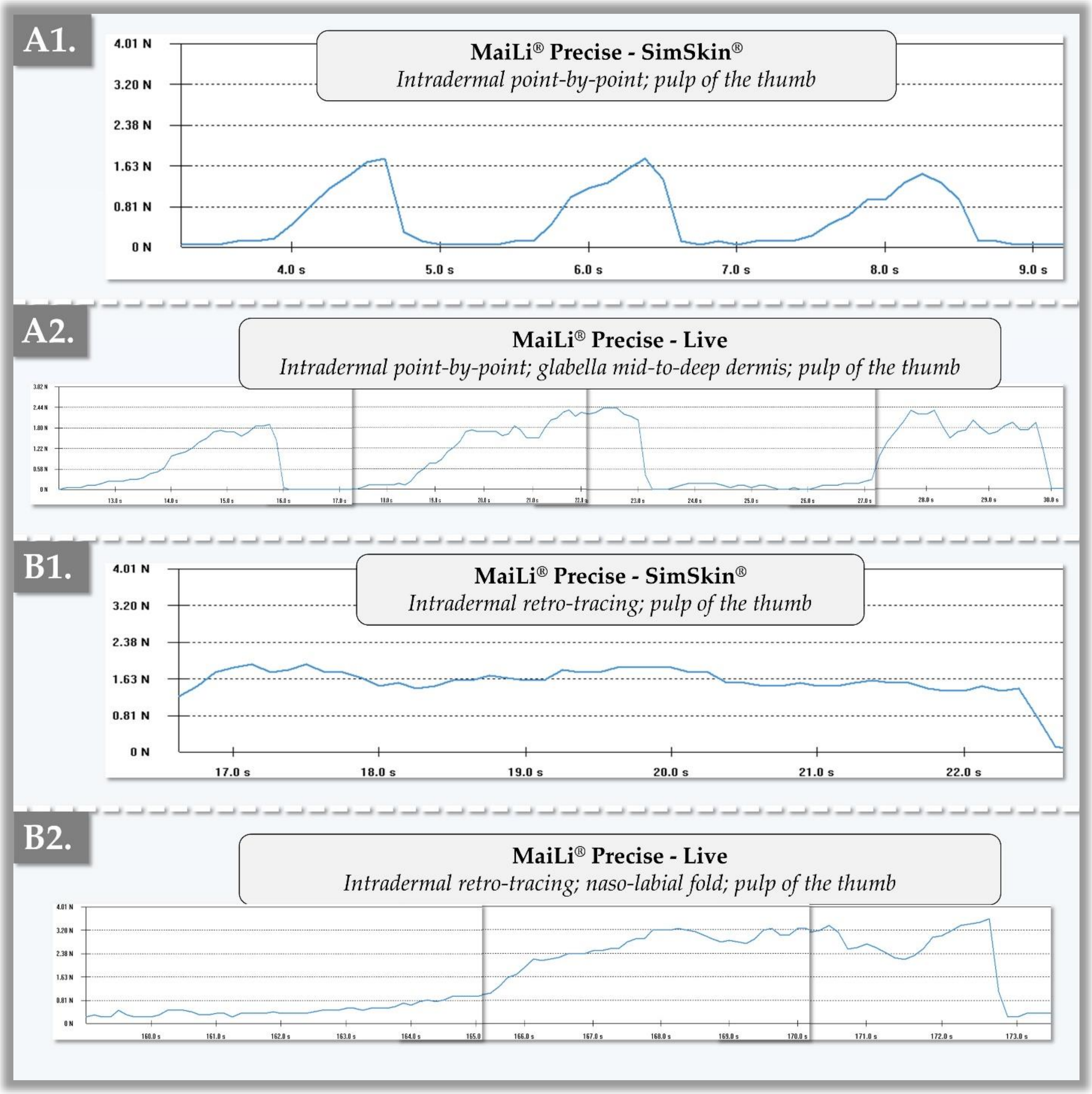

Figure S8. Injection force profiles of the MaiLi® Precise dermal filler during in vitro (**A1**,**B1**) and in vivo (**A2**,**B2**) injectability assessments (i.e., Participant N°1). N, Newtons.

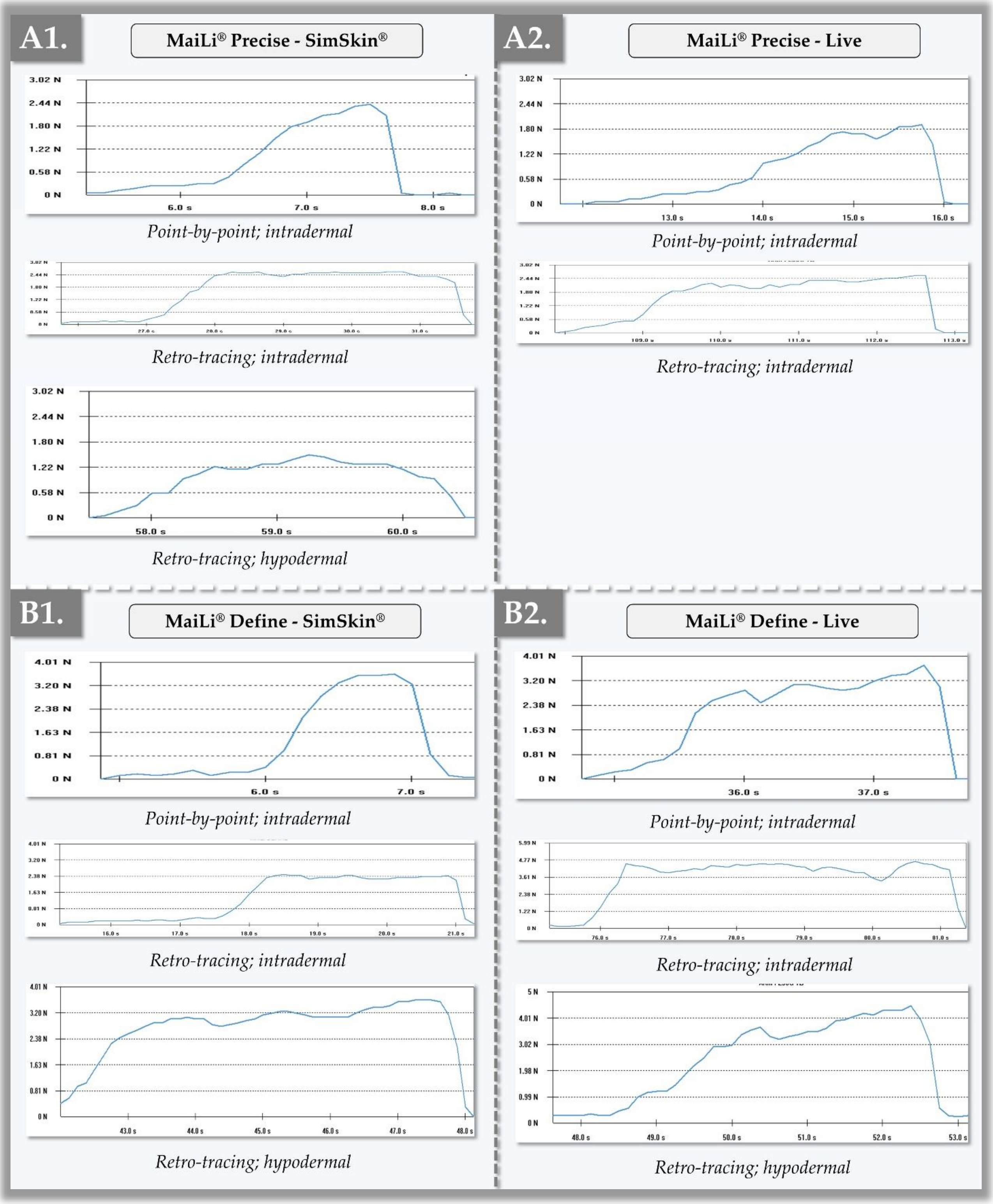

Figure S9. Injection force profiles of MaiLi® Precise (**A1**,**A2**) and MaiLi® Define (**B1**,**B2**) dermal fillers during in vitro and in vivo injectability assessments (i.e., Participants N°1 and N°2). N, Newtons.

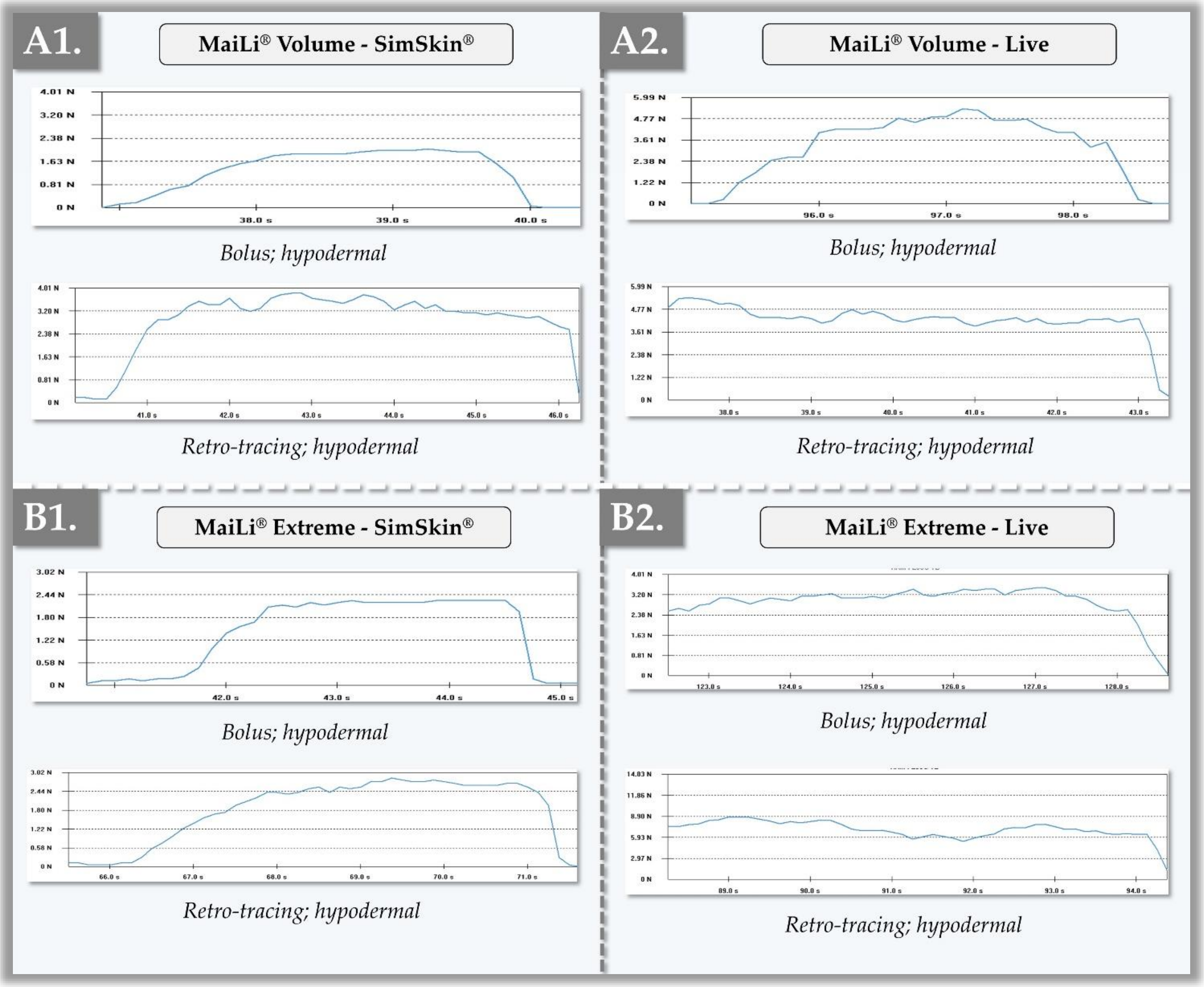

Figure S10. Injection force profiles of MaiLi® Volume (**A1**,**A2**) and MaiLi® Extreme (**B1**,**B2**) dermal fillers during in vitro and in vivo injectability assessments (i.e., Participants N°3 and N°4). N, Newtons.

2. Supplementary Tables

Table S1. Quantitative results of in vivo injection forces for the MaiLi® Precise dermal filler (i.e., Participant N°1). N, Newtons; N/A, non-applicable; PM, Patrick Micheels; TB, Thierry Bezzola.

Treated Area	Injection Technique	Injector	Thumb Area Used to Inject	Mean / Maximal / Low and High Forces Exerted on the Plunger Rod Hilt (N) [1]
Glabella	Point-by-point	PM	Pulp	Maximum applied force of 2.79 N (multiple points)
	Point-by-point	PM	P1-P2 joint	Maximum applied force of 3.31 N (multiple points)
Glabella	Point-by-point	TB	Pulp	Maximum applied force of 3.89 N (multiple points)
	Point-by-point	TB	P1-P2 joint	N/A
Nasolabial folds	Point-by-point	PM	Pulp	N/A
	Point-by-point	PM	P1-P2 joint	Maximum applied force of 3.13 to 3.31 N (multiple points)
	Retro-tracing	PM	Pulp	On the plateau, maximum force reached at 2.38 to 3.60 N
	Retro-tracing	PM	P1-P2 joint	On the plateau, maximum force reached at 3.61 to 5.87 N
	Antero-tracing	PM	Pulp	On the plateau, maximum force reached at 2.20 to 2.55 N
	Antero-tracing	PM	P1-P2 joint	N/A
Nasolabial folds	Point-by-point	TB	Pulp	Maximum force reached at 3.89 N
	Point-by-point	TB	P1-P2 joint	On the plateau, maximum force reached at 2.09 to 3.31 N
	Retro-tracing	TB	Pulp	On the plateau, maximum force reached at 6.22 N
	Retro-tracing	TB	P1-P2 joint	On the plateau, maximum force reached at 3.61 to 5.87 N
	Antero-tracing	TB	N/A	N/A

[1] Multiple points of injection are possible.

Table S2. Quantitative results of in vivo injection forces for the MaiLi® Define dermal filler (i.e., Participant N°2). N, Newtons; N/A, non-applicable; PM, Patrick Micheels; TB, Thierry Bezzola.

Treated Area	Injection Technique	Injector	Thumb Area Used to Inject	Mean / Maximal / Low and High Forces Exerted on the Plunger Rod Hilt (N) [1]
Glabella	Point-by-point	PM	Pulp	Maximum force applied of 1.45 to 1.51 N (multiple points)
	Point-by-point	PM	P1-P2 joint	N/A
Glabella	Point-by-point	TB	P1-P2 joint	N/A
Nasolabial folds	Point-by-point	PM	Pulp	Maximum force applied of 4.82 to 4.91 N (multiple points)
	Point-by-point	PM	P1-P2 joint	On the plateau, maximum force reached at 2.90 to 4.47 N
	Retro-tracing	PM	Pulp	On the plateau, maximum force reached at 2.90 to 4.47 N
	Retro-tracing	PM	P1-P2 joint	N/A
	Antero-tracing	PM	Pulp	On the plateau, maximum force reached at 3.31 to 4.70 N (multiple points)
	Antero-tracing	PM	P1-P2 joint	N/A
Nasolabial folds	Point-by-point	TB	Pulp	Maximum force applied of 5.11 N
	Point-by-point	TB	P1-P2 joint	Maximum force applied of 5.93 to 8.72 N (multiple points)
	Retro-tracing	TB	Pulp	Maximum force applied of 5.11 N
	Retro-tracing	TB	P1-P2 joint	On the plateau, maximum force reached at 5.87 to 11.33 N
	Antero-tracing	TB	N/A	N/A

[1] Multiple points of injection are possible.

Table S3. Quantitative results of in vivo injection forces for the MaiLi® Volume dermal filler (i.e., Participant N°3). N, Newtons; N/A, non-applicable; PM, Patrick Micheels; TB, Thierry Bezzola.

Treated Area	Injection Technique	Injector	Thumb Area Used to Inject	Mean / Maximal / Low and High Forces Exerted on the Plunger Rod Hilt (N) [1]
Nasolabial folds	Point-by-point	PM	Pulp	Maximum force applied of 5.29 N
	Point-by-point	PM	P1-P2 joint	On the plateau, maximum force reached at 6.70 to 7.67 N
	Point-by-point	TB	P1-P2 joint	N/A
Cheek hollows	Bolus	PM	Pulp	Maximum force applied of 2.09 N
	Bolus	PM	P1-P2 joint	N/A
	Retro-tracing	PM	Pulp	On the plateau, maximum force reached at 1.45 to 2.38 N
	Retro-tracing	PM	P1-P2 joint	N/A
Cheek hollows	Bolus	TB	Pulp	N/A
	Bolus	TB	P1-P2 joint	Maximum force applied of 11.86 N
	Retro-tracing	TB	Pulp	On the plateau, maximum force reached at 6.55 to 8.25 N
	Retro-tracing	TB	P1-P2 joint	On the plateau, maximum force reached at 9.18 to 13.95 N
Marionet	Point-by-point	PM	Pulp	N/A
	Point-by-point	PM	P1-P2 joint	Maximum force applied of 8.14 N
	Retro-tracing	PM	Pulp	On the plateau, maximum force reached at 1.22 to 1.33 N
	Retro-tracing	PM	P1-P2 joint	N/A
Marionet	Retro-tracing	TB	P1-P2 joint	N/A

[1] Multiple points of injection are possible.

Table S4. Quantitative results of in vivo injection forces for the MaiLi® Extreme dermal filler (i.e., Participant N°4). N, Newtons; N/A, non-applicable; PM, Patrick Micheels; TB, Thierry Bezzola.

Treated Area	Injection Technique	Injector	Thumb Area Used to Inject	Mean / Maximal / Low and High Forces Exerted on the Plunger Rod Hilt (N) [1]
Cheek bones	Bolus close-to-the-bone	PM	Pulp	On the plateau, maximum force reached at 2.67 to 2.96 N
	Bolus close-to-the-bone	PM	P1-P2 joint	On the plateau, maximum forces exceeded the dynamometer calibration (i.e., > 14.83 N)
Cheek bones	N/A	TB	N/A	N/A

[1] Multiple points of injection are possible.

Table S5. Quantitative results of in vivo injection forces for the MaiLi® Extreme dermal filler (i.e., Participant N°5). N, Newtons; N/A, non-applicable; PM, Patrick Micheels; TB, Thierry Bezzola.

Treated Area	Injection Technique	Injector	Thumb Area Used to Inject	Mean / Maximal / Low and High Forces Exerted on the Plunger Rod Hilt (N) [1]
Temples	Bolus in the fat (several injections)	PM	Pulp	On the plateau, maximum force reached at 3.60 to 4.10 N<br>Slow injection: On the plateau, maximum force reached at 1.27 to 1.80 N<br>Rapid injection: Maximum force reached at 3.19 to 6.22 N
	Bolus in the fat (several injections)	PM	P1-P2 joint	On the plateau, maximum forces varying from 5.69 to 8.72 N<br>Slow injection: On the plateau, maximum force reached at 6.22 to 10.11 N<br>Rapid injection: Maximum force reached at 12.50 N
Temples	N/A	TB	N/A	N/A
